# A Case Report of Switching from Specific Vendor-Based to R-Based Pipelines for Untargeted LC-MS Metabolomics

**DOI:** 10.3390/metabo10010028

**Published:** 2020-01-08

**Authors:** Álvaro Fernández-Ochoa, Rosa Quirantes-Piné, Isabel Borrás-Linares, María de la Luz Cádiz-Gurrea, PRECISESADS Clinical Consortium, Marta E. Alarcón Riquelme, Carl Brunius, Antonio Segura-Carretero

**Affiliations:** 1 Department of Analytical Chemistry, Faculty of Sciences, University of Granada, Av Fuentenueva s/n, 18071 Granada, Spain; ansegura@ugr.es; 2 Research and Development of Functional Food Centre (CIDAF), Health Science Technological Park, Av del Conocimiento, No. 37, s/n, 18016 Granada, Spain; rquirantes@cidaf.es (R.Q.-P.); iborras@cidaf.es (I.B.-L.); 3 Centre for Genomics and Oncological Research (GENYO), Pfizer-University of Granada-Andalusian Government, Health Science Technological Park, Av de la Ilustración 114, 18016 Granada, Spain; marta.alarcon@genyo.es; 4 Department of Biology and Biological Engineering, Chalmers University of Technology, SE-412 96 Gothenburg, Sweden

**Keywords:** metabolomics, data pre-processing, mass spectrometry, liquid chromatography, R packages, vendor software

## Abstract

Data pre-processing of the LC-MS data is a critical step in untargeted metabolomics studies in order to achieve correct biological interpretations. Several tools have been developed for pre-processing, and these can be classified into either commercial or open source software. This case report aims to compare two specific methodologies, Agilent Profinder vs. R pipeline, for a metabolomic study with a large number of samples. Specifically, 369 plasma samples were analyzed by HPLC-ESI-QTOF-MS. The collected data were pre-processed by both methodologies and later evaluated by several parameters (number of peaks, degree of missingness, quality of the peaks, degree of misalignments, and robustness in multivariate models). The vendor software was characterized by ease of use, friendly interface and good quality of the graphs. The open source methodology could more effectively correct the drifts due to between and within batch effects. In addition, the evaluated statistical methods achieved better classification results with higher parsimony for the open source methodology, indicating higher data quality. Although both methodologies have strengths and weaknesses, the open source methodology seems to be more appropriate for studies with a large number of samples mainly due to its higher capacity and versatility that allows combining different packages, functions, and methods in a single environment.

## 1. Introduction

Metabolomics is defined as the complete characterization of low molecular weight molecules (metabolites) present in a biological system, such as cells, tissues, biofluids, or organisms [1]. Untargeted metabolomics are frequently used to compare metabolic profiles between subjects to identify differences associated with the underlying study question (e.g., disease, diet, etc.) [2].

Untargeted metabolomics studies are carried out through a series of the following steps: (i) study design and sample recruitment, (ii) sample preparation, (iii) instrumental analysis, (iv) data pre-processing and statistical analysis, (v) compound identification, and (vi) biological interpretation [3,4]. These steps must be carried out with high precision and accuracy to maintain data quality throughout the pipeline that allows interpreting the results and to address the underlying biological issue of the study [5]. The analytical techniques most frequently used in this type of studies are proton Nuclear Magnetic Resonance Spectroscopy (^1^H-NMR) and Mass Spectrometry (MS). The main advantages of NMR are the high reproducibility/repeatability and accurate quantification, as well as capacity of structure elucidation. MS, on the other hand, is able to detect a much higher number of metabolites due to its higher sensitivity [6]. In addition, MS is usually coupled to various separation techniques at the front end, such as Liquid Chromatography (LC-MS). This hyphenation is able to separate the analytes prior to MS detection in order to achieve better MS performance. In metabolomics, LC-MS is the most employed analytical technique [7]. Among the different steps involved in untargeted metabolomic workflows using LC-MS, this research is primarily focused on data pre-processing. 

Data pre-processing of the LC-MS data is a critical step which involves reducing the complexity of the raw data, extracting the main features, and transforming them in order to subsequently perform adequate statistical tests [8]. This process encompasses a series of steps, such as baseline correction, noise filtering, peak detection, peak alignment, normalization, missing data imputation, and annotation [8,9,10]. Different vendor and open source software have been developed to perform these functions [11,12]. In this sense, the main commercial platforms at present correspond to the major instrument vendors: Mass Profinder/Profiler from Agilent Technologies (La Jolla, CA, USA) [13,14], Progenesis QI from Waters Corporation, Compound Discover from Thermo Scientific, MetaboScape from Brucker, and SIEVE from Thermo Scientific. On the other hand, open source software has gained in popularity in recent years [7]. Some highly popular software are MZmine [15], Workflow4Metabolomics [16], MetAlign [17], OpenMS [18], and XCMS [19]. Several, if not most, software modules are based on the programming language R [7], with a recent survey showing that the most used tool to pre-process LC-MS data is XCMS [20].

Ideally, the perfect platform to data processing in metabolomics should be intuitive with a user-friendly interface, open-source, and offer a comprehensive coverage of all steps (or at least with easy integration to other steps) of the pipeline [7]. While commercial software stands out for being intuitive and user-friendly, open source solutions are free to use and provide more versatility to the needs of the users. However, in general they are also less intuitive and have steeper learning curves [7]. Moreover, it is also quite common that different tools show great effectiveness in some of the data processing steps but not in others. Users therefore have to stitch together different tools to carry out the entire pre-processing pipeline which often demands more advanced bioinformatics and/or programming skills [8]. As there are a lot of tools for metabolomics data processing, there is a need to compare these methodologies and to examine their pros or cons [7,21].

In the present research, two specific methodologies, Mass Profinder/Profiler from Agilent Technologies vs. a pipeline based on four R packages (IPO [22], XCMS [19], batchCorr [23], and RAMClustR [24]) for data pre-processing in untargeted metabolomics studies were compared. We highlighted differences in these two pipelines using 369 plasma samples analyzed by LC-MS, from a dataset aimed to investigate the metabolism of Systemic Autoimmune Diseases within the PRECISESADS project (http://www.precisesads.eu/). The software from Agilent Company was selected as model of vendor software for comparison since the LC-MS equipment used was acquired from that commercial company. Our aim was to provide insights into benefits and disadvantages of using these two methodologies, thereby aiding metabolomics researchers in their choice of data pre-processing strategies as well as proposing tools for switching from vendor-based to open source pipelines for these types of studies. Although tutorials may exist for individual pre-processing modules, tutorials on how to stitch together modules into entire pipelines are lacking. In this way, a detailed tutorial on how to start using an R-based methodology is provided to offer users with outlines from which to build their own custom pre-processing pipelines.

## 2. Results and Discussion

Starting from the idea that data pre-processing is a critical step to decrease the risk of chance findings and misinterpretation and achieve correct biological interpretations, we have compared two specific pre-processing pipelines, Agilent software and a methodology based on R packages.

Due to the large number of samples, the methodology based on Profinder software was not able to perform the data processing of all samples in a single step due to the capacity of the computer. Consequently, batch recursive feature extraction had to be performed separately for five different subsets of the entire data set. In contrast, the R-based methodology allowed pre-processing of all samples at once, and depending on the number of available computer cores, the pre-processing would be more or less fast.

In the next subsections, we present and discuss several results obtained from the two methodologies, i.e., number of peaks, degree of missingness, quality of the peaks, degree of misalignments, and robustness in multivariate models.

### 2.1. Peak Picking

After grouping of features likely arising from the same metabolite (merging of isotopes, adducts, and fragments) both methodologies obtained a similar number of molecular features (Agilent methodology: 548, R-based methodology: 531) and degrees of missing data (Agilent methodology: 8.91%; R-based methodology: 9.59%). The molecular features were cross-checked by retention time (RT) and *m/z*, and, in total, 445 were picked by both methodologies. Nevertheless, when using LC-MS techniques, several thousand features are detected in the biospecimen analyzed. However, in the present case, only 531/548 molecular features were detected. This happened because the noise level was set high (1000 counts) in order to ensure that all molecular features detected correspond to biological molecules. In previous tests with a lower noise level, we were able to detect a higher number of features (≈1000) but the signals with low intensity presented difficulties for biological identification. Therefore, the noise level was increased up to 1000 counts for better comparison of the two methodologies.

Regarding the molecular features that were not extracted by any of the mentioned methodologies, these were explored in raw data. Some examples of these peaks are shown in Figure S1 (Supplementary Material). Most of these features were characterized by the absence of a clear Gaussian peak shape or the presence of double peaks very close in retention times, mainly due to isomeric structures. As the peak search algorithms are different between both methodologies, these molecular features were not extracted by any of them for the mentioned reasons. In addition, another difference between both methodologies corresponds to the variable filtering step according to RSD. Filtering was performed after or during the normalization step, in the vendor and R-based methodologies, respectively. These differences in the filtering step could produce that some features would just be filtered by only one of the two methodologies.

RT drift was well aligned by XCMS, which modifies the RT for the samples to achieve superpositioning of the chromatograms (Figure S2, Supplementary Material). In contrast, the Mass Hunter Profinder software does not modify the RT of the chromatograms, but instead, it tries to find the features in the samples within a RT range. With the high number of injections, RT drifts were pronounced, resulting in poor peak matching for several features. Those failures need to be corrected by the operator one by one, being a very time-consuming step. An example of this type of failure is shown in Figure S3 (Supplementary Material). Since manual supervision and correction of the results is highly time consuming, an advantage of the Profinder software is the ease of visualizing the molecular features. Manual inspections and corrections, are, however, much more tedious in the R-based approach. XCMS integrations were therefore indirectly assessed by Pearson correlation with peak areas obtained from the Agilent workflow after manual inspection and correction, (Figure S4, Supplementary Material), which showed overall a very high accordance. Interestingly, peak area correlations decreased somewhat when comparing XCMS peaks to those obtained from the Agilent software prior to manual correction (Figure S5, Supplementary Material), suggesting better results obtained by using R-based pipeline in tremens of alignment and integration. We hypothesize that this could be highly related to the greater number of parameters that can be modified and optimized. In contrast, the used vendor software does not allow adjusting so many parameters and there is no automatic optimization process.

### 2.2. Normalization Results

**Figure 1:**
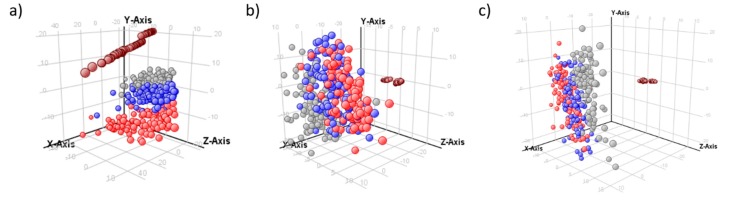
PCA scores plot from data obtained by Agilent MassHunter Profinder software. (**a**) Raw data; (**b**) data normalized by the quantile method (Mass Profiler Professional, MPP); (**c**) data normalized by the percentile shift (75.0) method (MPP); batch 1 in red, batch 2 in blue, batch 3 in gray, and QCs in brown.

**Figure 2:**
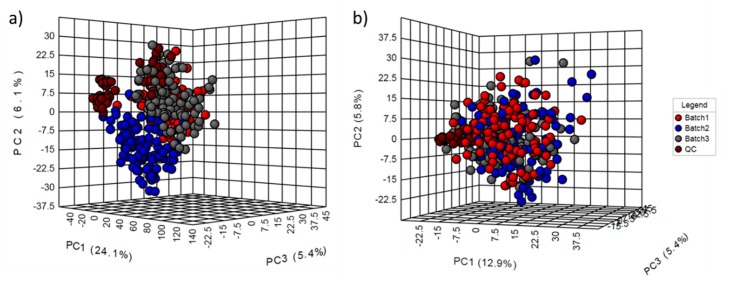
PCA scores plot from data obtained by open source methodology. (**a**) Raw data; (**b**) data normalized by the batchCorr package (R environment); batch 1 in red, batch 2 in blue, batch 3 in gray, and QCs in brown.

The metabolomic data from the three batches was collected in different months and each batch lasted for about a week. These facts produced large between-batch and within-batch effects. The magnitude of these drifts was detected by the distribution of the QC samples in the PCA score plots [4] from raw data obtained with both Agilent MassHunter Profinder software (Figure 1a) and R-based (Figure 2a) methodologies. These effects are quite common in large-scale LC-MS studies due to different reasons, such as matrix effects, variations in chromatographic conditions, loss of mass ionization efficiency, or variability in MS sensibility [25]. Consequently, normalization is one of the most critical steps in any pre-processing pipelines, to ensure that the data is comparable, without losing valuable biological information [26]. The number of normalization methods in vendor software is in general limited. Specifically, in Agilent Mass Profiler Professional software (MPP), the offered methods are by internal standards, quantile and percentile shift. Normalization by internal standards is widely considered to be not fit for purpose in untargeted metabolomics [27]. The other techniques did not provide satisfactory normalization and showed that study samples were visibly separated by the batch (Figure 1). These normalization methods are based on the signal intensity distributions [28] and do not consider possible feature drift patterns [23]. In order to improve the obtained results by vendor software, data was also normalized by the open access platform MetaboAnalyst 4.0, which showed improved efficacy (Figure S6, Supplementary Material). Furthermore, MetaboAnalyst has the advantages that it is both free to use and has a friendly, intuitive web-based interface (https://www.metaboanalyst.ca/). However, it is also important to note that this tool is mainly oriented to statistical analysis and not pre-processing.

Unlike Agilent MPP software, there are several open source programs based on R to carry out the normalization step in large untargeted LC-MS metabolomics studies, such as MetNormalizer [27], BatchCorr [23], MixNorm [29], Normalyzer [30], or NormalizeMets [31], among others. Most of them are based on QC samples taking into consideration the possible feature drift patterns. In this way, the open source package (bathCorr) applied to our data showed good results getting a well-behaved grouping of the QC samples and allowed the batch effects to correct in a higher degree (Figure 2). The main advantage of batchCorr is that it takes into account different possible drift trends along the sequence [23], and examples of some of these different patterns are shown in Figure S7 (Supplementary Material). Therefore, different correction functions are used depending on the detected drifts. However, as an example of the less thought through user experience in most R packages, the native PCA plots provided by the batchCorr package were very rudimentary (Figure S8, Supplementary Material). Nevertheless, it is important to clarify that the low resolution of the graphics obtained by batchCorr is not generalizable to all developed packages based on R. The data were therefore imported in MetaboAnalyst to obtain more visually pleasing figures (Figure 2).

### 2.3. Multivariate Models

**Figure 3:**
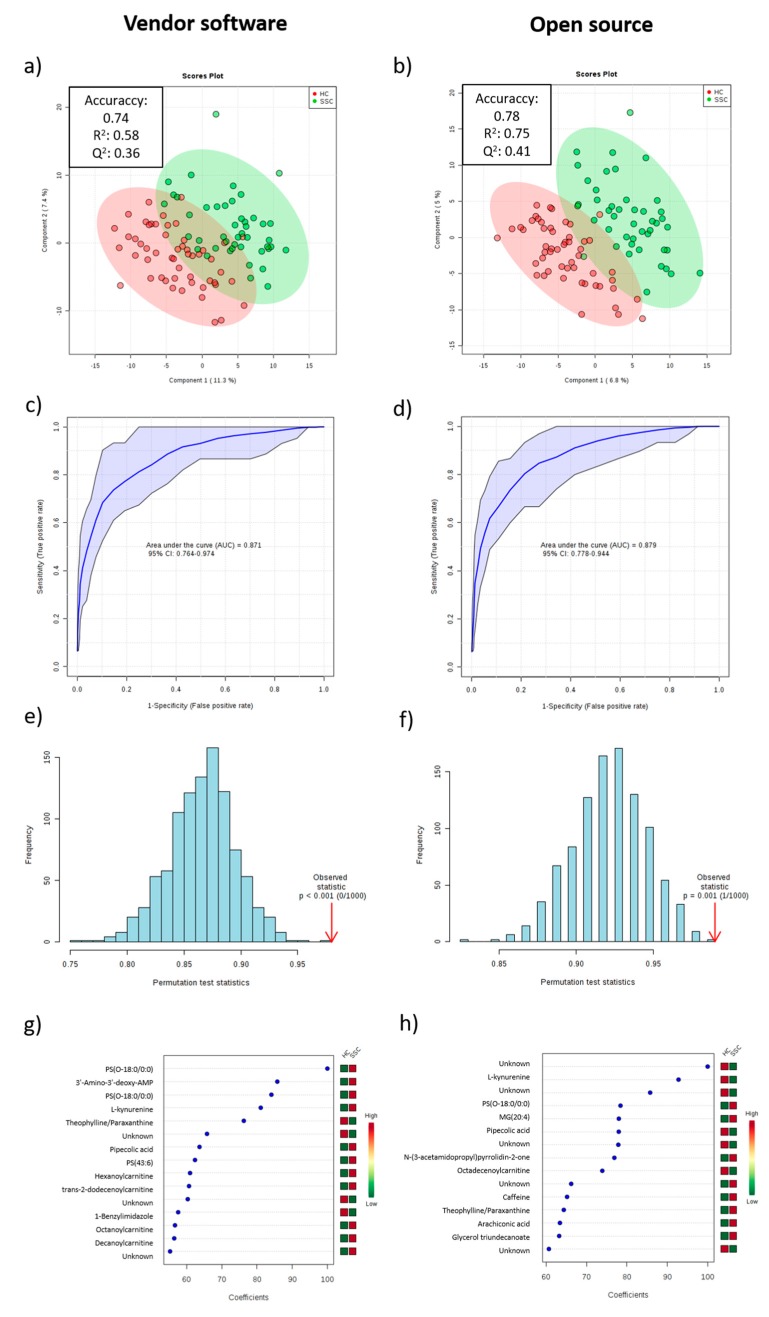
A supervised Partial Least Squares Discriminant Analysis (PLS-DA) performed in the MetaboAnalyst 4.0 software. PLS-DA scores plot (**a**), Agilent Profinder Software Data (V), (**b**), open source data (O), ROC curve for PLS-DA model validation (**c**), V; (**d**), O, permutation test result (**e**), V; (**f**), O, 15 most significant features (**g**), V; (**h**), O.

A subset of samples (systemic sclerosis patients and healthy controls) was selected for multivariate modeling. The same statistical tests were performed for the data using both methodologies. First, PLS-DA models performed by MetaboAnalyst 4.0 showed slightly higher classification accuracy and predictive power using data obtained from the R pipeline (Figure 3). More detailed information on the top-ranked metabolite features (Figure 3g,h) are available in the Supplementary Material (Tables S1 and S2). Six metabolites (L-kynurenine, PS(18:0), Pipecolic acid, Theophylline, and two unknowns) were found among the 15 most important in both PLS-DA models. Although only these six compounds were common in both PLS-DA models, several of the other signals appeared between positions 15 and 30 of the VIP ranking of the opposite model (Table S1). Another factor is related to the different number of molecular features used for the PLS-DA models (548 vs. 531). Therefore, some of the metabolites were not statistically significant in both PLS-DA models since they were only extracted by one methodology. In this way, the PLS-DA models were also performed using only the 445 common molecular features. In these models, 11 molecular features were common among the 15 variables with higher VIP values. The results of these PLS-DA models are shown in Figure S9 (Supplementary material). According to the previous models, better classification results were also obtained by making use of the data pre-processed by the R-based methodology.

**Figure 4:**
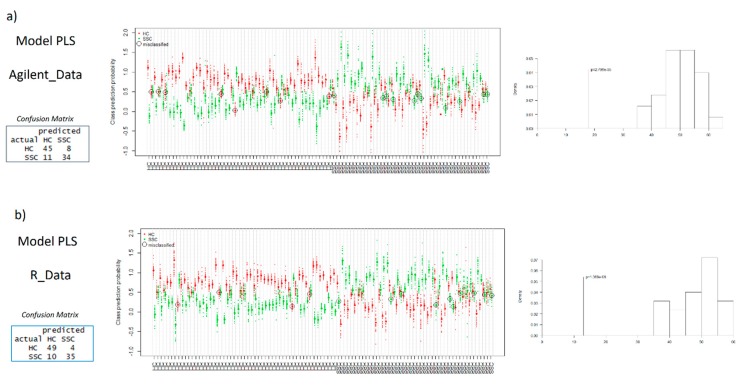
Confusion matrices, permutation test results, and predictive classification of individuals according to PLS results obtained using MUVR package. (**a**) Profinder software data, (**b**) open source data.

PLS models were also performed in R using the MUVR package, which employs a more prudent cross-validation scheme (repeated double cross-validation) and also performs unbiased variable selection [32]. Analogously to the PLS analyses performed using MetaboAnalyst, slightly better classifying results were found with the data obtained in R (Table 1). Overall, better modeling results were obtained for the R data, including parsimony, represented by a lower number of selected variables. Misclassifications and the confusion matrices are shown in Figure 4, and complete lists with annotated metabolites are provided in the Supplemental Tables S3 and S4. The higher number of components and variables in the model with data from Profinder software may make the biological interpretation of the results more difficult [33]. In addition, the ideal model would be the one that achieves better classifying results with a smaller number of variables. Therefore, the better results obtained with R data indicate higher data quality compared to the commercial pre-processing pipeline.

In view of the annotated metabolites, L-kynurenine and phosphatidylserine (PS) were found among the most significant variables in all four multivariate models. The kynurenine pathway has shown a large impact in recent years due to its relation with the immune system, inflammation and neurological processes [34].

Furthermore, the dysregulation of the kynurenine pathway is in agreement with results from other autoimmune diseases, such as systemic lupus erythematosus (SLE) [35].

Other differential metabolites in the majority of the models were acylcarnitines, unsaturated fatty acids (UFAs), and phospholipids. The dyregulation of these metabolites, mainly acylcarnitines and UFAs, are in line with previous research on a smaller number of volunteers [36], which gives consistency to the data obtained by both methodologies in this subset of samples.

### 2.4. Global Comparison of Both Methodologies

Based on the results obtained in the previous sections, the main advantages and disadvantages of each methodology are highlighted in Table 2.

The Agilent software methodology is characterized by its ease of use, a high level of dedicated support, and good integration with annotation modules. In view of the results obtained, commercial software seems to be appropriate for studies of metabolomics with a smaller number of samples, where there is little drift in *m/z*, RT, or signal intensity over time. However, for metabolomics studies with a larger number of samples, as in the case of the example shown, the commercial software used have limitations mainly in the capacity to process a high number of samples as well as in correcting for signal drifts. In addition, the high occurrence of incorrect peak integration requires extensive efforts by researchers for manual correction. Fortunately, these disadvantages can be addressed using open source methodology, e.g., in R, although this environment is not as user friendly or intuitive as commercial software. Furthermore, if the user has never worked with R-based methodologies, the initial learning curve is very steep. To compensate for this difficulty and to aid R beginners in setting up a data pre-processing and analytical pipeline, a tutorial is provided in the online Supplementary materials.

Candidate biomarkers discovery should ideally be independent on the methodology used for data processing [11]. However, we have shown differences in the selection of candidate metabolites obtained by the two different methodologies in the presented example related to Systemic Sclerosis. In fact, multivariate models had a higher classification rate and were more parsimonious using data obtained by the open source R methodology. These observed differences are likely related to the quality of the data used to create such models. In view of the results, the differences in data quality can be highly influenced predominantly by the normalization step, which has been revealed as e main weakness of the vendor software methodology.

## 3. Materials and Methods

### 3.1. Dataset

Metabolomic data were obtained from samples of the PRECISESADS project (www.precisesads.eu). The aim of this project is to find clinically useful biomarkers in order to obtain a new reclassification of 7 systemic autoimmune diseases (systemic lupus erythematous, rheumatoid arthritis, systemic sclerosis, mixed connective tissue disease, antiphospholipid syndrome, Sjögren’s syndrome, and undifferentiated connective tissue disease). This metabolomic analysis is ancillary to the written informed consent obtained from each participant of the study, which was registered on clinicaltrials.gov with the code NCT02890121.

Plasma samples from 247 patients with the above diseases and 59 healthy volunteers were analyzed. Subjects were recruited from different study centers across Europe. Biological samples were obtained and stored at −80 °C until analysis. A Quality Control (QC) sample was obtained by mixing 20 µl of each study sample including both controls and case samples. After thawing on ice, a protein precipitation step was carried out with a mix of methanol-ethanol (1:1; v/v). Samples were analyzed using an Agilent 1260 HPLC instrument coupled to an Agilent 6540 Ultra High Definition (UHD) Accurate Mass Q-TOF equipped with a Jet Stream dual ESI interface. Metabolites were separated using a reversed-phase C18 analytical column (Agilent Zorbax Eclipse Plus, 3.5 μm, 2.1 × 150 mm) and detected in positive-ion mode over a range from 50 to 1700 *m*/*z*. The analytical methodology is described in detail elsewhere [36].

The QC sample was injected five times at the beginning of each sequence in order to stabilize the equipment and also continuously throughout the analytical sequence (each five study samples) to monitor system performance and perform feature intensity drift correction. Due to the large number of samples, instrumental analysis was performed in three batches. In addition, MS/MS analysis of the QC sample was performed in order to obtain a representative fragmentation pattern of the main metabolites present in the majority of the samples. This analysis was carried out using nitrogen as the collision gas with 10 eV, 20 eV, and 40 eV as collision energies.

### 3.2. Data Pre-Processing

**Figure 5:**
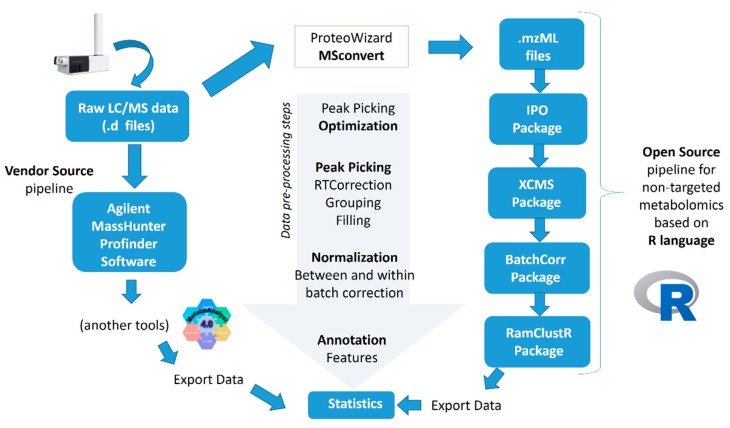
A summary scheme of both methodologies (Agilent software vs. R-based pipeline) used in the comparative study.

Figure 5 schematically shows a summary of both methodologies carried out for pre-processing of the data. Both methodologies are described in detail in the following subsections.

#### 3.2.1. Agilent MassHunter Profinder Software Approach

Data was processed using the *Agilent MassHunter Profinder B.06.00* software using Automatic peak finding by the two-step method. This software was installed on a Windows 7 computer with 3.20 GHz Intel Core i7 and 32 GB of RAM memory.

First, a batch recursive feature extraction was performed using data from QC samples as a representative sample in which all endogenous metabolites should be present. Due to the large number of sample files and their size, molecular feature extraction of the QC files was performed in the first place. Second, the molecular features found in the QC samples were then used to guide feature selection in the case and control study samples. In this step, peaks with intensity lower than 1000 counts were filtered out. Isotopes and adducts were grouped into a molecular feature with a maximum charge of 2. Feature alignment was performed with 20 ppm ± 2 mDa mass and 0.25 min retention time windows. Molecular features were manually inspected and corrected before integration. Both percentile shift and quantile normalization methods were tested using the Mass Profiler Professional software (Agilent Technologies). Due to the large between-batch and within-batch effects, the data were normalized using two methods consecutively. Firstly, the Bayes method from MetaboAnalyst 4.0 [37,38,39], and secondly, the Mass Total Useful Signal (MSTUS) method. Finally, the molecular features with high variability in QC samples (relative standard deviation, RSD, higher than 30%) were removed.

#### 3.2.2. R-Based Approach

First, Agilent .d files were converted to .mzML file format using the *MSConvertGUI* software [40] to be able to import them into the R open source environment (version 3.5.1). The R scripts, packages, and commands were applied in RStudio environment (version 1.1.456) to facilitate use and visualization of the results.

The *XCMS* package was used for peak picking retention time alignment, grouping, and filling of missing features [19]. XCMS parameters were optimized using a combination of the *IPO* package [22] and manual optimization. For IPO optimization, 6 QC files spanning the multi-batch injections sequence were selected. The final optimized parameters for peak picking using the “centwave” method were the following: peakwidth = c (12.45, 35), mzdiff = 0.00175, prefilter = c (3, 1000). Retention time adjustment was performed with the “obiwarp” method using the following optimized values: profStep = 0.3, response = 13.84, gapInit = 0.352, gapExtend = 2.436. Finally, feature correspondence was achieved with the “density” method using the following optimized parameters: bw = 5.0 and mzwid = 0.047.

Imputation of values still missing after XCMS peak filling was performed using an in-house script based on RandomForest (https://gitlab.com/CarlBrunius/StatTools; mvImpWrap function). The obtained data was corrected for within- and between-batch intensity drift using the *batchCorr* package [23]. Moreover, the features with high variability after normalization (RSD > 30%) were filtered out.

Finally, grouping of features (isotopes, adducts, and fragments) corresponding to the same metabolites was achieved using the RAMClustR package [24]. RAMClust grouping is based on similarity between features in retention time and intensity correlations between samples. The similarity parameters (σ_t_, σ_r_) were optimized using an in-house procedure and were set at values of 1.33 and 0.3, respectively.

All R scripts used in this research are available in full detail with comments as a tutorial in the Supplementary materials.

### 3.3. Statistics and Metabolite Annotation

In order to compare both methodologies, different statistical tests were performed. The Pearson correlation test was used to study the similarity of metabolite features obtained with both methodologies. Moreover, a subset of samples (53 healthy control and 45 patients with systemic sclerosis) was chosen for multivariate data analysis. PLS-DA models were performed using MetaboAnalyst 4.0 [41] and the R MUVR package [32]. Permutation tests were performed in both models for validation [42].

To provide biological meaning to the results, metabolites of interest were annotated according to Metabolomics Standard Initiative (MSI) guidelines [43]. Annotation was performed by comparing MS and MS/MS spectra with information from metabolomics databases (LipidMaps, KEGG Human Metabolome Database and METLIN) as well as MS/MS fragmentation resources, such as MetFrag and Sirius [44,45].

## 4. Conclusions and Future Research

Both vendor and open source methodologies have strength and weaknesses. However, we have shown that the open source methodology is the most suitable option for metabolomic studies with a larger number of samples in multiple batches. First, this methodology is to a much higher degree able to correct the large between- and within-batch effects. In addition, it stands out for being free and open source, having a greater capacity and versatility to use a large number of packages, functions, and methods in a single environment. Nevertheless, this environment is also less intuitive, frequently with lower quality graphical output, and with a distinctly steeper learning curve. We provide a detailed tutorial to help users of commercial software to start processing data through R-based methodology. Nevertheless, our study has some limitations related to the possibility of generalizing the results to the rest of commercial software. It is important to recognize that each of the vendor software has its own advantages and disadvantages that may differ from the commercial software used, Agilent MassHunter Profinder. Future research should therefore focus on comparing other vendor software with the proposed R-based pipeline.

## Figures and Tables

**Table 1:** Main results (number of variables (nVar), classification rate (class, %), Area Under the Curve (AUC), number of components (nComp), and *p*-value of permutations test) obtained for the PLS models using MUVR package.

PLS-MUVR Models	nVar	Class (%)	AUC	nComp	*p*-Value
R Data	15	86.8	0.931	2	1.38 × 10^−6^
Profinder software Data	67	81.7	0.893	3	2.80 × 10^−5^

**Table 2:** Main advantages and disadvantages of the use of Profinder software and R packages (IPO, XCMS, batchCorr, and RamClustR) for pre-processing of metabolomics data obtained by HPLC-ESI-QTOF-MS.

Profinder Software Methodology		R-Based Methodology	
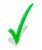	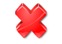	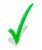	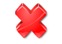
Easy to use, user-friendly interface	License fee	Open source	Steep learning curve
High quality of the plots	Limited capacity to process a high number of samples	Greater number of packages, functions, and methods (e.g., normalization)	Low plot quality (plots obtained with the specific R packages used)
No need to transform the format of the data	Few normalization techniques. Difficulties to normalize large between-batch effects	High capacity for faster processing of a high number of samples	Data format transformation
Easy to inspect features, integration results, and MS spectra. Easy to predict molecular formula	Errors in peak integration	Possibility of carrying out all the steps of pre-processing and statistical analysis in the same environment	More cumbersome to show integration results, MS spectra, and to predict molecular formula
Easy to manually correct areas	Low control of the processing (only some parameters can be modified)	Flexibility and versatility	Some level of coding skills is required

## Supplementary Materials

The following are available online at https://www.mdpi.com/2218-1989/10/1/28/s1. (Supplementary material 1) and (Supplementary material 2-A brief Tutorial on R-based approach).
