# Supplementary material for: A Case Report of Switching from Specific Vendor-Based to R-Based Pipelines for Untargeted LC-MS Metabolomics
Source: Metabolites. 2020 Jan 8;10(1):28. doi: 10.3390/metabo10010028 (PMC7022532; doi:10.3390/metabo10010028)
Supplement: Supplementary file 1 [file metabolites-10-00028-s001.zip › A brief Tutorial on Rvf.pdf]

A brief Tutorial on *R-based approach*

Costs and benefits of switching from vendor-based to open source pipelines for untargeted LC-MS metabolomics.

This tutorial aims to detail step-by-step how the R approach has been developed. We pretend to show the way we have applied the different R packages in our data. In this sense, our main objective is to help beginners in R language to be able to use the different packages and scripts used in the manuscript. Although tutorials may exist for individual pre-processing modules, in this document is showed how to stitch together these modules into entire pipelines. Nevertheless, we recommend you reading the different manuscripts and tutorials of each R package available in the following links:

IPO (Libiseller et al. 2015):
https://bioconductor.org/packages/release/bioc/vignettes/IPO/inst/doc/IPO.html
XCMS (Smith et al. 2006):
https://bioconductor.org/packages/release/bioc/vignettes/xcms/inst/doc/xcms.html
https://jotsetung.github.io/metabolomics2018/xcms-preprocessing.html
batchCorr (Brunius et al. 2016):
https://gitlab.com/CarlBrunius/batchCorr/tree/master/Tutorial
RAMClustR (Broeckling et al. 2014):
http://pubs.acs.org/doi/abs/10.1021/ac501530d
MUVR (Shi et al. 2018):
https://github.com/CarlBrunius/MUVR/blob/master/README.md

1) Installation.

First, R and RStudio were downloaded from the websites (https://www.r-project.org/, https://www.rstudio.com/) and installed on a Windows 7 computer. Second, the different packages used were installed in the R environment with the following codes:

```
install.packages("BiocManager", repos="http://cran.us.r-project.org",
dependencies=TRUE)
install.packages("devtools", repos="http://cran.us.r-project.org",
dependencies=TRUE)
```

```
library(devtools)
library(BiocManager)

BiocManager::install("IPO", version = "3.8")
BiocManager::install("xcms", version = "3.8")
install_github("cbroeckl/RAMClustR", build_vignettes = TRUE, dependencies
= TRUE)
devtools::install_git("https://gitlab.com/CarlBrunius/batchCorr.git")
```

```
library(IPO)
library(xcms)
library(RAMClustR)
library(batchCorr)
```

In order to be able to read the data in the R environment, it is necessary to transform the data format into an adequate format (mzML, mzXML, mzData, NetCDF). In our particular case, data were collected by an Agilent instrument in a .d format. We transformed this format into .mzML using *MSConvertGUI* software. This tool from proteowizard can be downloaded from the website [http://proteowizard.sourceforge.net/download.html.](http://proteowizard.sourceforge.net/download.html) This software is easy to use as it is shown schematically in the following figure.

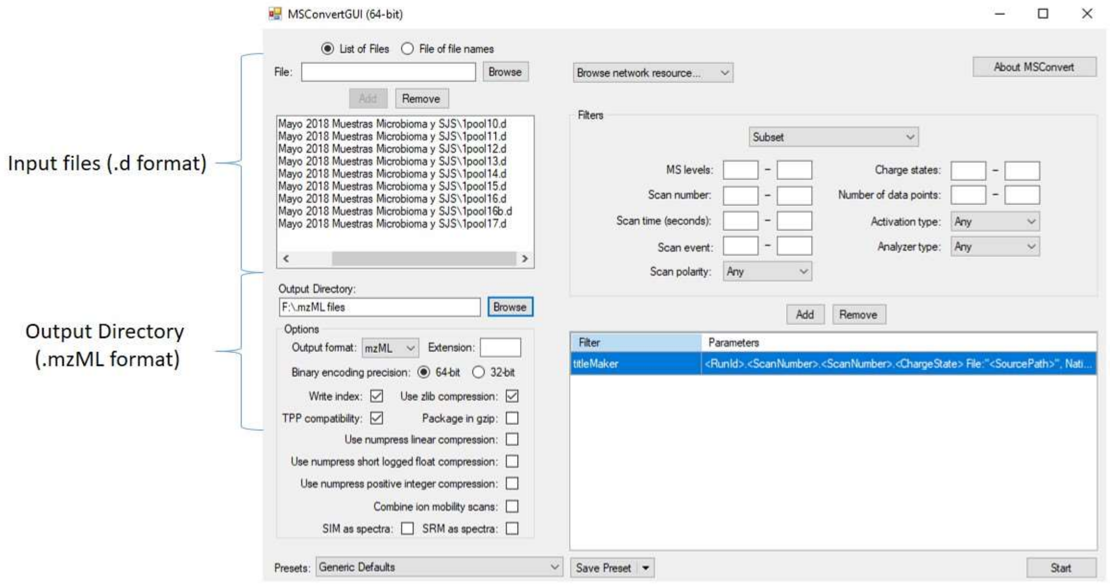

2) IPO.

The aim of this package is to optimize the xcms parameters. For this purpose, 6 QC files well distributed throughout the sequence were selected.

```
library(xcms)
library(RColorBrewer)
library(pander)
library(magrittr)
library("IPO", lib.loc="~/R/win-library/3.5")
library("msdata", lib.loc="~/R/win-library/3.5")
```

The selected files must be saved in a folder within the working directory. If you do not know what this directory is, use the command “>getwd()”. In our case, the 6 QC files were located in a folder called “QC” and we used the following command to import them into the R environment.

```
#Open files
datafiles <- list.files("QC", recursive = TRUE, full.names = TRUE, pattern
=".mzML")
peakpickingParameters <- getDefaultXcmsSetStartingParams('centWave')
```

To work more quickly in R, it is very useful to work in parallel using the different computer cores. For this, we use the “doParallel” package. In order to continue working with the computer while the R commands are executing, a good number of cores is the numbers of available cores minus one.

```
library(doParallel)
nCore=detectCores()-1
```

Next, the different parameters for peak peaking were selected. As our data were obtained by a high resolution mass spectrometer, the peak picking method was ‘CentWave’. Some parameters were optimized by IPO package selecting a reasonable range according to our experiments. In this way, *min_peakwidth*, *max_peakwidth* and *ppm* were the optimized parameters. However, the *noise* and *prefilter* parameters were set at a single value. These parameters were selected after observing the current noise level in the chromatograms. The selection of the different parameters was performed with the following code:

```
#PeakPickingParameters
peakpickingParameters <- getDefaultXcmsSetStartingParams('centWave')
peakpickingParameters$noise=1000
peakpickingParameters$value_of_prefilter=(3,800)
peakpickingParameters$min_peakwidth<- c(8,15)
peakpickingParameters$max_peakwidth<- c(30,40)
peakpickingParameters$ppm<- c(25,35)
param=SnowParam(workers = 5)
```

Once the parameters were selected, the optimization of the parameters set in a range was performed by IPO.

```
resultPeakpicking <-
  optimizeXcmsSet(files = datafiles,
                  params = peakpickingParameters,
                  BPPARAM = param,
                  nSlaves = 1,
                  subdir = NULL,
                  plot = TRUE)
```

The IPO optimization is based on Design of Experiments. The results were shown both numerically and graphically in contour graphs.

```
#best parameter settings:
#  min_peakwidth: 12.48
#max_peakwidth: 35
#ppm: 24
#mzdiff: 0.00175
#snthresh: 10
#noise: 1000
#prefilter: 3
#value_of_prefilter: 800
#mzCenterFun: wMean
#integrate: 1
#fitgauss: FALSE
#verbose.columns: FALSE
```

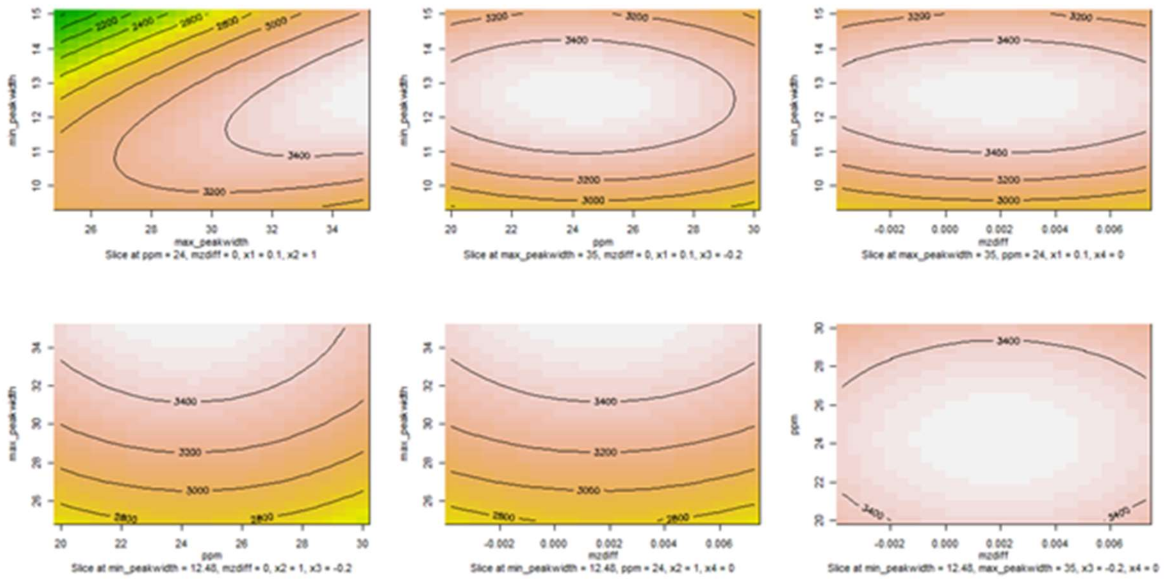

Analogously to the peak picking optimization, the retention time alignment and grouping parameters were also optimized in order to be applying in XCMS package. The optimized

parameters for retention time alignment using the “obiwarp” method were *profStep*, *response*, *gapInit* and *gapExtend*, and for correspondence using “density” method were *bw* and *mzwid.* In our study, the parameter *minfrac* was fixed at value of 0.5.

```
optimizedXcmsSetObject <- resultPeakpicking$best_settings$xset

retcorGroupParameters <- getDefaultRetGroupStartingParams()
retcorGroupParameters$profStep <- c(0.33,1)
retcorGroupParameters$gapExtend <- c(2.1,2.9)
retcorGroupParameters$minfrac=0.5
retcorGroupParameters$response=c(9.6,17.6)
retcorGroupParameters$gapInit=c(0.14, 0.54)
retcorGroupParameters$mzwid=c(0.023, 0.047)

BiocParallel::register(BiocParallel::SerialParam())

resultRetcorGroup <-
  optimizeRetGroup(xset = optimizedXcmsSetObject,
                   params = retcorGroupParameters,
                   nSlaves = 1,
                   subdir = NULL,
                   plot = TRUE)
```

The results were obtained by the following code and these are shown below:

```
writeRScript(resultPeakpicking$best_settings$parameters, resultRetcorGroup
$best_settings)
xset <- retcor(
xset,
method         = "obiwarp",
plottype       = "none",
distFunc       = "cor_opt",
profStep       = 0.3,
center         = 6,
response       = 13.84,
gapInit        = 0.352,
gapExtend      = 2.436,
factorDiag     = 2,
factorGap      = 1,
localAlignment = 0)
xset <- group(
xset,
method  = "density",
bw      = 0.879999999999999,
mzwid   = 0.047,
minfrac = 0.5,
minsamp = 1,
max     = 50)
```

Despite the optimization made by IPO, the obtained value for *bw* was considered very low. For this reason, this parameter was optimized manually. Different tests were performed with different values of bw (from 0.5 to 10). The optimal parameter of bw was chosen when the

greatest number of features was obtained. In our case, it was obtained at 5.0 as it is showed in the next figure.

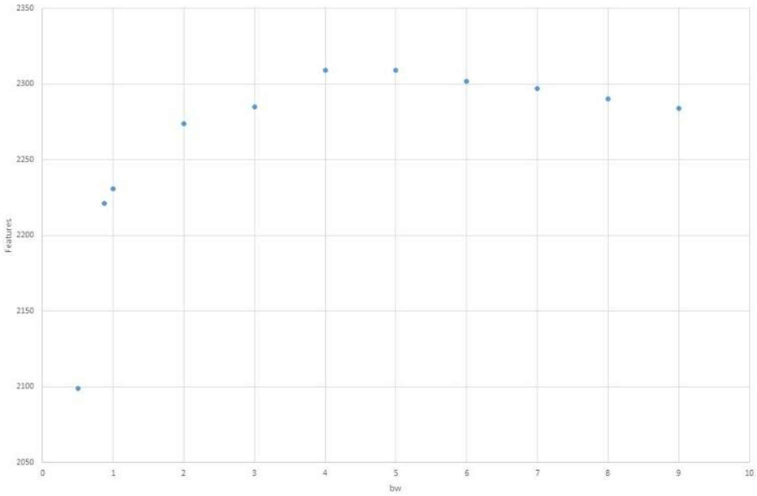

3) XCMS

Before using XCMS, we imported all data in R analogously to the importation performed previously with the 6 QC samples for the IPO optimization. Thus, we pasted the .mzML files of all the samples in a folder called "PlasmaData" within the workspace and run the following code.

```
datafiles <- list.files("PlasmaData", recursive = TRUE, full.names = TRUE,
pattern=".mzML")
```

The samples were divided in two subfolders in order to create a phenodata data.frame depending on if they are cases or QC samples.

```
# Create a phenodata data.frame
pd <- data.frame(sample_name = sub(basename(datafiles), pattern = ".mzML",
                                   replacement = "", fixed = TRUE),
                 sample_group = c(rep("Case", 306), rep("QC", 63)),
                 stringsAsFactors = FALSE)
```

The peak picking, retention time correction, and feature correspondence were performed using XCMS with the parameters optimized in the previous step with IPO.

```
raw_data <- readMSData(files = datafiles, pdata = new("NAnnotatedDataFrame
", pd), mode = "onDisk")

#PeakPicking Step
```

```
cwp <- CentWaveParam(peakwidth = c(12.48, 35), ppm = 24, mzdiff = 0.00175,
snthresh = 10, noise = 1000, prefilter = c(3,800))
xdataL1 <- findChromPeaks(raw_data, param = cwp)

[...Detecting chromatographic peaks in 2519 regions of interest ... OK: 17
55 found.
Detecting mass traces at 24 ppm ... OK
Detecting chromatographic peaks in 2574 regions of interest ... OK: 1825 f
ound.
Detecting mass traces at 24 ppm ... OK
Detecting chromatographic peaks in 2634 regions of interest ... OK: 1849 f
ound. ...]
```

#Grouping and RT Correction

```
#RT Correction

BiocParallel::register(BiocParallel::SerialParam())
xdataL2 <- adjustRtime(xdataL1, param = ObiwarpParam(gapInit = 0.352, gapE
xtend = 2.436, response = 13.84, binSize = 0.3))

[...Sample number 185 used as center sample.
Aligning 0601429.mzML against 3920435.mzML ... OK
Aligning 0610035.mzML against 3920435.mzML ... ]


#Correspondence
pdp <- PeakDensityParam(sampleGroups = xdataL2$sample_group, minFraction =
0.5, bw = 5, binSize = 0.047)

xdataL3 <- groupChromPeaks(xdataL2, param = pdp)
Processing 67195 mz slices ... OK
```

The next step is to look for the peaks which were assigned as missing values (NA) in some samples because the peak detection algorithm was not able to find them. Therefore, this step of "filling peaks" is performed with the following code.

```
xdataL4 <- fillChromPeaks(xdataL3)
[Defining peak areas for filling-in .... OK
Start integrating peak areas from original files
Requesting 488 missing peaks from 0601429.mzML ... got 394.
Requesting 622 missing peaks from 0610035.mzML ... got 510.
Requesting 553 missing peaks from 0611111.mzML ... got 393.
...]
```

Despite this new step, there are still missing values in the samples for different reasons (e.g. not present in the biological samples, very low concentrations below the detection limits, algorithm failures). It is shown below the total number of missing values of all samples before and after the filling step.

```
#Number of missing values before filling step
xdataL3b = featureValues(xdataL3, value = "into")
sum(is.na(xdataL3b))
[1] 136846
#Number of missing values after filling step
xdataL4b = featureValues(xdataL4, value = "into")
sum(is.na(xdataL4b))
```

```
[1] 43476
```

Once these steps have been performed, we proceed to extract the integrated areas and the information of the peaks (mass and retention times) with the following codes. In this step, the features are named according to their mass and retention times using the "featureDefinitions" function as follows.

```
xdataL5 = featureValues(xdataL4, value = "into")
xdataL5= t(xdataL5)
featData=featureDefinitions(xdataL4)
featData=featData@listData
featNames=paste0(featData$mzmed,"_",featData$rtmed)
colnames(xdataL5)=featNames
```

For example, the first feature (mz: 100.075826364437, rt: 273.94580078125 s) was called "100.075826364437_273.94580078125".

Missing value imputation

Imputation of values still missing after XCMS peak filling was performed using an in-house script based on RandomForest methodology (https://gitlab.com/CarlBrunius/StatTools; mvImpWrap() function). For it, it is necessary to install firstly the *StatTools* package.

```
#Installation of package StatTools
devtools::install_git("https://gitlab.com/CarlBrunius/StatTools.git")

#Missing Value Imputation
nCore=detectCores()-1
cl=makeCluster(nCore)
registerDoParallel(cl)
Imp <- StatTools::mvImpWrap(MAT = xdataL5, method = "RF")
xdataL5 <- Imp
stopCluster(cl)

#Number of missing values after missing value imputation
sum(is.na(xdataL5))
[1] 0
```

4) BatchCorr

The BatchCorr package aims to normalize the data due to between-batch and within-batch drifts produced in LC-MS analysis. This normalization strategy is based on the QC samples,

the batches, and the injection order. For these reasons, it is necessary to introduce this information (injection, batch, and QC/Case) in the R environment.

Previously, a dataframe called "pd" was created with the information of the kind of sample (QC or Case sample). Therefore, we needed to add in this dataframe two columns to indicate the injection position and the batch of each sample.

First of all, it is necessary to read a .csv file with the information of injection order and batch of each sample. This file (in our case is named "pdinjbatch.csv") have to be located in the workspace.

sample_name	sample_group	batch	inj
pool1-r005	QC	1	1
4315311	Case	1	2
4315335	Case	1	3
4315359	Case	1	4
4315383	Case	1	5
4315211	Case	1	6
pool2	QC	1	7
4315235	Case	1	8
4315259	Case	1	9
4315283	Case	1	10
617211	Case	1	11
617235	Case	1	12
pool3	QC	1	13
617259	Case	1	14
617283	Case	1	15
3499535	Case	1	16
3499559	Case	1	17
3499583	Case	1	18

```
pd_injbatch = read.csv(file='pd_injbatch.csv', head = TRUE, sep = ',')
```

Once this file was loaded in the R environment, the columns "inj" and "batch" were added to the dataframe *pd*. For this step, it is needed to make sure that the samples are sorted in both files (pd and pd_injbatch) in the same way. Below are the instructions to carry out this stage:

```
# Check the identical order of the samples.
rownames(pd) = pd$sample_name
rownames(pd_injbatch) = pd_injbatch$sample_name
identical(rownames(pd), rownames(pd_injbatch))
[1] TRUE

# Add the "inj" and "batch" columns to the dataframe pd.
pd<- data.frame(pd, inj= pd_injbatch$inj)
pd<- data.frame(pd, batch= pd_injbatch$batch)
```

To perform the *batchCorr* package correctly is required that the samples are sorted by their injection order. If they are not ordered in the previous files in this way, it is mandatory to apply the following commands.

```
xdataL5Sort= xdataL5[order(pd$inj),]
pdSort = pd[order(pd$inj),]
```

The normalization by BatchCorr is performed by means of three steps: 1: between-batch correspondence/alignment. 2: within-batch intensity drift correction and 3: between-batch normalization. In the following lines are detailed how we used this package in our data. To better understanding the scripts and functions, we strongly recommend that you read the tutorial of this package (https://gitlab.com/CarlBrunius/batchCorr/tree/master/Tutorial)

In order to carry out these 3 steps, we needed these 3 objects: a) dataframe (pd) with information about batches, sample groups (QC/case) and injection orders; b) peak table without missing values (xdataL5Sort); c) peak table with missing values obtained before filling step. In order to obtain this last peak table (xdataL3bSort), we applied the following code:

```
xdataL3b = featureValues(xdataL3, value = "into")
xdataL3b= t(xdataL3b)
featData=featureDefinitions(xdataL4)
featData=featData@listData
featNames=paste0(featData$mzmed,"_",featData$rtmed)
colnames(xdataL3b)=featNames
xdataL3bSort=xdataL3b[order(pd$inj),]
```

Step 1. Between-batch correspondence/alignment

The aim of the first step is to align the features misaligned between batches. Firstly, it is fundamental to extract the retention times and masses from each feature using the function “peakInfo”. As our features were called, “mass_rt”, we applied the function as follows:

```
peakIn <- peakInfo(PT = xdataL3bSort, sep = '_', start = 1)
```

	mz	rt
feature_1	100.0758	273.94580
feature_2	100.1120	200.78700
feature_3	101.0791	273.85098
feature_4	102.0547	89.85000
feature_5	103.0541	304.76999
feature_6	104.0522	89.84962
feature_7	104.1061	1494.32019

Secondly, we used the "alignBatches" function to carry out the purpose of this first step.

```
alignBat <- alignBatches(peakInfo = peakIn, PeakTabNoFill = xdataL3bSort,
PeakTabFilled = xdataL5Sort, batches = pdSort$batch, sampleGroups = pdSort
$grp, selectGroup = 'QC')
```

IMPORTANT NOTIFICATION

```
There are no alignment candidates. Therefore,
  between-batch alignment is not possible.
Consider expanding mzdiff and/or rtdiff
  of that correspondence is accurate between batches.
Returning NULL.
```

This output indicates that no alignment candidates were found and, consequently, that no between-batch alignment was possible. This fact reflects that this stage was carried out correctly in the previous step with XCMS package.

Step 2. Within-batch intensity drift correction

The second step aims to normalize and correct the drifts produced throughout each batch. In addition, the features with a RSD higher than 30% in QC samples after the normalization were filtered.

We used the functions "getbatch" and "correctDrift" to extract each batch, and carry out the normalization, respectively.

```
batchB_F <- getBatch(peakTable = xdataL5Sort, meta = pdSort, batch = pdSor
t$batch, select = '1')

batch1 <- correctDrift(peakTable = batchB_F$peakTable, injections = batchB
_F$meta$inj, sampleGroups = batchB_F$meta$sample_group, QCID = 'QC', G = s
eq(5,35,by=3), modelNames = c('VVE', 'VEE'))#
```

```
    __  ___________    __  _____________
   /  |/  / ____/ /   / / / / ___/_  __/
  / /|_/ / /   / /   / / / /\__ \ / /
 / /  / / /___/ /___/ /_/ /___/ // /
/_/  /_/\____/_____/\____//____//_/    version 5.4.2
Type 'citation("mclust")' for citing this R package in publications.

Mclust fitting ...
  |=======================================================================
=========================================================================|
100%

MClust final model with 14 clusters and VEE geometry.
BIC performed in 14.54 seconds and clustering in 0.91 seconds.

Calculation of QC drift profiles performed.

Drift correction of 12 out of 14 clusters using QC samples only.
Corrected peak table in $TestFeatsCorr

Filtering by QC CV < 0.3 -> 1205 features out of 1312 kept in the peak tab
le.
Peak table in $TestFeatsFinal, final variables in $finalVars and cluster i
nfo in $actionInfo.
```

```
# Batch 2
batchA_F <- getBatch(peakTable = xdataL5Sort, meta = pdSort, batch = pdSor
t$batch, select = '2')

batch2 <- correctDrift(peakTable = batchA_F$peakTable, injections = batchA
_F$meta$inj, sampleGroups = batchA_F$meta$sample_group, QCID = 'QC', G = s
eq(5,35,by=3), modelNames = c('VVE', 'VEE'))#

Mclust fitting ...
  |=======================================================================
=======================================================================|
100%

MClust final model with 14 clusters and VEE geometry.
BIC performed in 24.26 seconds and clustering in 1.25 seconds.

Calculation of QC drift profiles performed.

Drift correction of 13 out of 14 clusters using QC samples only.
Corrected peak table in $TestFeatsCorr

Filtering by QC CV < 0.3 -> 1110 features out of 1312 kept in the peak tab
le.
Peak table in $TestFeatsFinal, final variables in $finalVars and cluster i
nfo in $actionInfo.

# Batch 3
batchH_F <- getBatch(peakTable = xdataL5Sort, meta = pdSort, batch = pdSor
t$batch, select = '3')

batch3 <- correctDrift(peakTable = batchH_F$peakTable, injections = batchH
_F$meta$inj, sampleGroups = batchH_F$meta$sample_group, QCID = 'QC', G = s
eq(5,35,by=3),modelNames = c('VVE', 'VEE'))


Mclust fitting ...
  |=======================================================================
=======================================================================|
100%

MClust final model with 8 clusters and VEE geometry.
BIC performed in 10.59 seconds and clustering in 0.22 seconds.

Calculation of QC drift profiles performed.

Drift correction of 4 out of 8 clusters using QC samples only.
Corrected peak table in $TestFeatsCorr

Filtering by QC CV < 0.3 -> 701 features out of 1312 kept in the peak tabl
e.
Peak table in $TestFeatsFinal, final variables in $finalVars and cluster i
nfo in $actionInfo.
```

Step 3. Between-batch normalization

The third step aims to merge the drift-corrected data (mergeBatches() function) and normalize the effects between batches (normalizeBatches() function).

```
mergedData <- mergeBatches(list(batch1, batch2, batch3))
normData <- normalizeBatches(peakTable = mergedData$peakTable, batches = p
dSort$batch, sampleGroup = pdSort$sample_group, population = 'all')
```

Once these functions were carried out, we extracted the peakTable (features and intensities) and exported it in a .csv file.

```
PTnorm <- normData$peakTable
write.csv(PTnorm, file="PTnorm_F.csv")
```

A good way to observe how the data has been transformed with this package is using Principal Component Analysis (PCA). In the following lines, it is shown the code for PCA analysis used for the three data sets (raw data, data after within-batch normalization step and data after between-batch normalization step).

```
pca1 <- prcomp(x = xdataL5Sort, center = T, scale. = T)
pca2 <- prcomp(x = mergedData$peakTable, center = T, scale. = T)
pca3 <- prcomp(x = PTnorm, center = T, scale. = T)
```

It is shown below the code to create the graphs that show the scores plots of each PCA model.

```
par(mfrow=c(1,3))
plot(pca1$x[,2:3], col=pdSort$batch, main = 'raw')
plot(pca2$x[,2:3], col=pdSort$batch, main = 'within')
plot(pca3$x[,2:3], col=pdSort$batch, main = 'between')
```

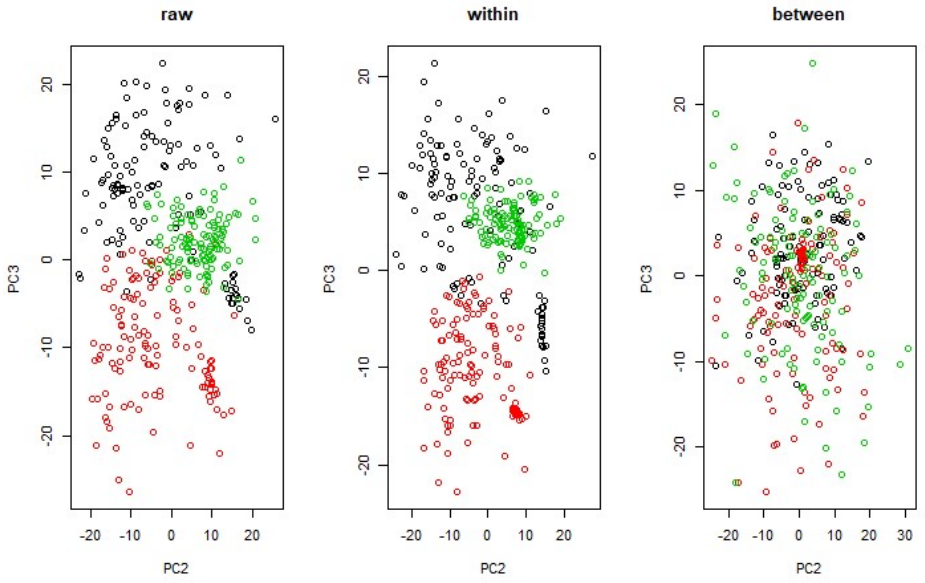

5) RAMClust

The aim of this package is to perform the annotation step. Briefly, it consists of grouping all MS signals related to a single compound (isotopes, adducts, isomers, fragments products, etc.) into a single file called "feature". To perform this step, the package RAMClust is mainly based on two parameters: (i) similarity in their retention times (st) and (ii) correlation in the abundances across different samples (sr).

```
library(RAMClustR)
```

Unlike the IPO package that allows optimizing the parameters for XCMS, there is still no package to optimize the mentioned RAMClust parameters, st and sr. So, we created our own methods and functions to optimize these values and achieve a better result of the annotation. In this way, the functions *getRamSt* and *plotClust* were created (see **Annex**).

The *getRamSt* function aims to obtain the optimal value of the **st** parameter.

```
getRamSt(xdataL4)
[1] 1.33
```

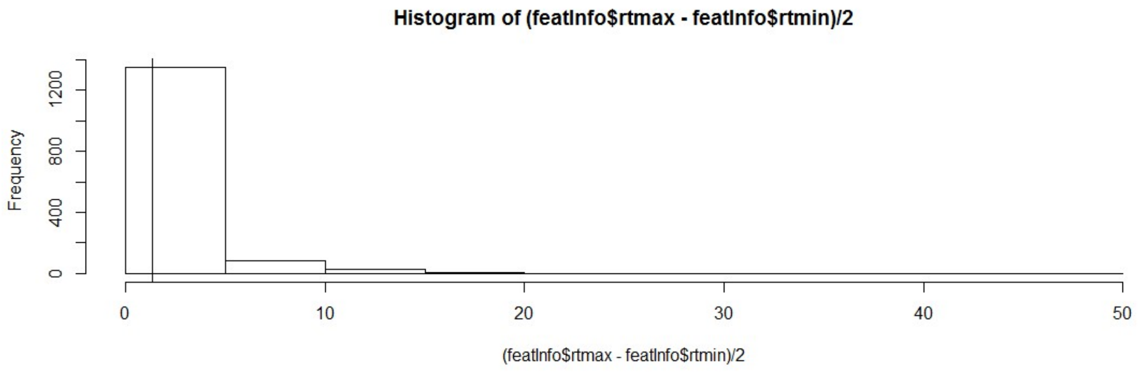

Once the st parameter was optimized, we applied the `ramclustR` function with this st value and a range of sr parameter (sr = 0.3, 0.4, 0.5). In this script, we used the plotClust function to obtain the plots of 20 features in order to observe the groupings of the signals and obtain the optimal sr value.

```
expDes=defineExperiment(force.skip = T)
sr=c(.3,.4,.5)
st=1.33
maxt=c(5)
par=expand.grid(st=st,sr=sr,maxt=maxt)
str(par)
'data.frame':   3 obs. of  3 variables:
 $ st  : num  1.33 1.33 1.33
 $ sr  : num  0.3 0.4 0.5
```

```
 $ maxt: num  5 5 5
 - attr(*, "out.attrs")=List of 2
  ..$ dim     : Named int  1 3 1
  .. ..- attr(*, "names")= chr  "st" "sr" "maxt"
  ..$ dimnames:List of 3
  .. ..$ st  : chr "st=1.33"
  .. ..$ sr  : chr  "sr=0.3" "sr=0.4" "sr=0.5"
  .. ..$ maxt: chr "maxt=5"
```

```
nClust=nSing=sizeMax=sizeMed=sizeMean=numeric(nrow(par))
nFeat=list()
samps=sample(1:205,20)
register(bpstart(SnowParam(7))) # Choose as many cores as you can/want
for (i in 1:nrow(par)) {
  RRP=ramclustR(ms='PTnorm_F.csv', st = par$st[i], sr=par$sr[i], maxt = pa
r$maxt, timepos = 2, sampNameCol = 1, featdelim = '_', ExpDes = expDes)
  nClust[i]=length(RRP$cmpd)
  nSing[i]=RRP$nsing
  sizeMax[i]=max(RRP$nfeat)
  sizeMed[i]=median(RRP$nfeat)
  sizeMean[i]=mean(RRP$nfeat)
  nFeat[[i]]=RRP$nfeat
  pdf(file=paste0('clusts_par',i,'.pdf'),width=15,height=8)
  par(mfrow=c(4,5),mar=c(4,4,2,0)+.5)
  clusts=round(c(2:6,seq(7,max(RRP$featclus),length.out = 15)))
  for (c in clusts) {
    plotClust(ram = RRP,clustnr = c,xcmsData = xdataL4,samps = samps)
  }
  dev.off()
}
```

Then, the results were obtained for each combination of values. To choose the optimal sr value, we visually inspected the generated files. In the following figure, examples of correct or incorrect annotation cases are shown. In this way, the optimized sr value was chosen where the greatest number of features with a correct annotation was obtained, (a value of 0.3 was obtained in our case).

```
cbind(par,nClust,nSing,sizeMax,sizeMean,sizeMed)
    st  sr maxt nClust nSing sizeMax sizeMean sizeMed
1 1.33 0.3    5    213   306      16 3.778302       3
2 1.33 0.4    5    213   257      17 4.009434       3
3 1.33 0.5    5    204   217      19 4.384236       3
```

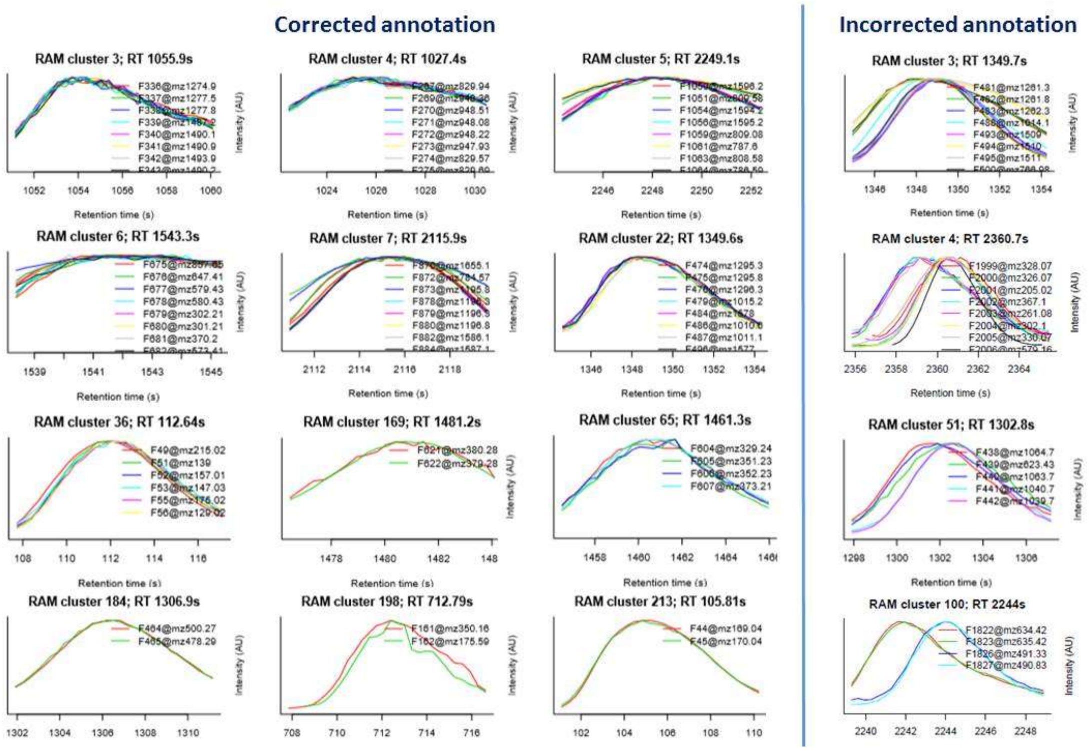

Finally, we applied the ramclustR function with the optimal values of st and sr.

```
RC1 <- ramclustR(ms='PTnorm_F.csv',
                 featdelim = "_",
                 st = 1.33,
                 sr = 0.3,
                 ExpDes=expDes,
                 sampNameCol = 1)
  organizing dataset
  normalizing dataset
calculating ramclustR similarity: nblocks =  1
1  RAMClust feature similarity matrix calculated and stored:
RAMClust distances converted to distance object
fastcluster based clustering complete
dynamicTreeCut based pruning complete
RAMClust has condensed 1109 features into 213 spectra
collapsing feature into spectral signal intensities
writing msp formatted spectra
msp file complete
```

```
RC1 <- do.findmain(RC1, mode = "positive", mzabs.error = 0.02, ppm.error =
10)
10 of 213
20 of 213
30 of 213
40 of 213
50 of 213
60 of 213
70 of 213
80 of 213
90 of 213
100 of 213
110 of 213
```

```
120 of 213
130 of 213
140 of 213
150 of 213
160 of 213
170 of 213
180 of 213
190 of 213
200 of 213
210 of 213
plotting findmain annotation results
finished
```

6) Export Data.

The last step of pre-processing corresponds to the export of data to use them in statistical analyses. We exported both the features annotated by RAMClustR and the signals that were not assigned to any cluster (singletons). In the following lines it is showed how the exportation was performed. The tidyverse package is necessary.

```
library(tidyverse)
```

#Look for the ion with the highest intensity in each group obtained by RAMClustR

```
Max_int<- lapply(RC1$M.ann, function(x) x[which.max(x$int), ])
Molecular_ions <- bind_rows(Max_int)
Molecular_ions$name <- paste(round(Molecular_ions$mz,4), round(RC1$clrt,2)
, sep = "_")
PTnorm_F2_mz <- colnames(PTnorm_F2)[-1] %>% str_split(.,"\\_") %>% lapply(
.,function(x) x[1]) %>% unlist() %>%as.numeric()
```

#check if it works for your data with the following table. The number of TRUES should be the same to the cluster number.

```
PTnorm_F2_mz %in% Molecular_ions$mz %>% table()
FALSE  TRUE
  896   213

PTnorm_F2_molecularIons_rawInt<- PTnorm_F2[,-1][,PTnorm_F2_mz %in% Molecul
ar_ions$mz]
```

#Look for the singletons

```
clustered_mz<- lapply(RC1$M.ann, function (x) x$mz) %>% unlist() %>% as.nu
meric()
singletons <- PTnorm_F2[,-1][,!PTnorm_F2_mz%in%clustered_mz]
```

#Combine singletons and molecular ions

```
PTnorm_F2_ramclust_annotedPT <- cbind.data.frame(PTnorm_F2_molecularIons_r
awInt, singletons)
```

```
rownames(PTnorm_F2_ramclust_annotedPT) <- paste0("PTnorm_F2",rownames(PTnorm_F2_ramclust_annotedPT))

colnames(PTnorm_F2_ramclust_annotedPT) <- paste0("PTnorm_F2",colnames(PTnorm_F2_ramclust_annotedPT))

write.csv(PTnorm_F2_ramclust_annotedPT, file = "PTnorm_F2_ramclust_annotedPT0920.csv")
```

Finally, a .csv file (samples vs intensities of each feature) was obtained in the working directory. This can be imported into different statistical software to perform the corresponding tests that are desired.

We hope this guide allows you to use R in the same way that we have used it in our work.

Good luck and thanks for your interest in this tutorial.

Álvaro Fernández and Carl Brunius

PS. Please don't hesitate to contact us if you have problems with the codes and functions or any suggestions or questions.

alvaroferochoa@ugr.es

carl.brunius@chalmers.se

Annex

getRamSt:

```
getRamSt <- function(XObj) {
  featInfo <- featureDefinitions(XObj)
  hist((featInfo$rtmax-featInfo$rtmin)/2)
  st <- round(median(featInfo$rtmax-featInfo$rtmin)/2, digits = 2)
  abline(v=st)
  return(st)
}
```

plotClust:

```
plotClust=function(ram,clustnr,xcmsData,samps,dtime=5,dmz=.05) {
  if(missing(samps)) {
    nSamp=nrow(ram$SpecAbund)
    samps=1:nSamp
  } else nSamp=length(samps)
  whichFeats=which(ram$featclus==clustnr)
  peakMeta=cbind(ram$fmz,ram$frt)
  pkMetaGrp=peakMeta[whichFeats,]
  rtr=ram$clrt[clustnr]+c(-dtime,dtime)
  rtr[rtr<0]=0
  mzr=cbind(ram$fmz[whichFeats]-dmz,ram$fmz[whichFeats]+dmz)
  chr <- chromatogram(xcmsData, mz = mzr, rt = rtr)
  plot(0:1,0:1,type='n',axes=F,xlab='Retention time (s)', ylab='Intensity
(AU)',main=paste0('RAM cluster ',clustnr,'; RT ',signif(ram$clrt[clustnr],
5),'s'))
  box(bty='l')
  for (pk in 1:length(whichFeats)) {
    rts=ints=list()
    for (samp in 1:nSamp) {
      rts[[samp]]=chr[pk,samps[samp]]@rtime
      ints[[samp]]=chr[pk,samps[samp]]@intensity
    }
    nrts=min(sapply(rts,length))
    rts=sapply(rts,function(x) x[1:nrts])
    rts=rowMeans(rts)
    ints=sapply(ints,function(x) x[1:nrts])
    ints=rowMeans(ints,na.rm=T)
    par(new=T)
    plot(rts,ints,type='l',col=pk+1,ylim=c(0,max(ints,na.rm=T)),axes=F,xla
b='',ylab='')
  }
  axis(1)
  legend('topright',legend = paste0('F',whichFeats,'@mz',signif(pkMetaGrp[
,1],5)), lty=1,col=(1:length(whichFeats))+1,bty='n')
}
```
