# Supplementary material for: A Case Report of Switching from Specific Vendor-Based to R-Based Pipelines for Untargeted LC-MS Metabolomics
Source: Metabolites. 2020 Jan 8;10(1):28. doi: 10.3390/metabo10010028 (PMC7022532; doi:10.3390/metabo10010028)
Supplement: Supplementary file 1 [file metabolites-10-00028-s001.zip › Supplementary MaterialR2v2.pdf]

Supplementary material

Costs and benefits of switching from vendor-based to open source pipelines for untargeted LC-MS metabolomics.

Fig.S1. Examples of peaks (explored in raw data) that were extracted by one of the two methodologies. A) Molecular features extracted by Agilent Profinder software. B) Molecular features extracted by the R-based pipeline.

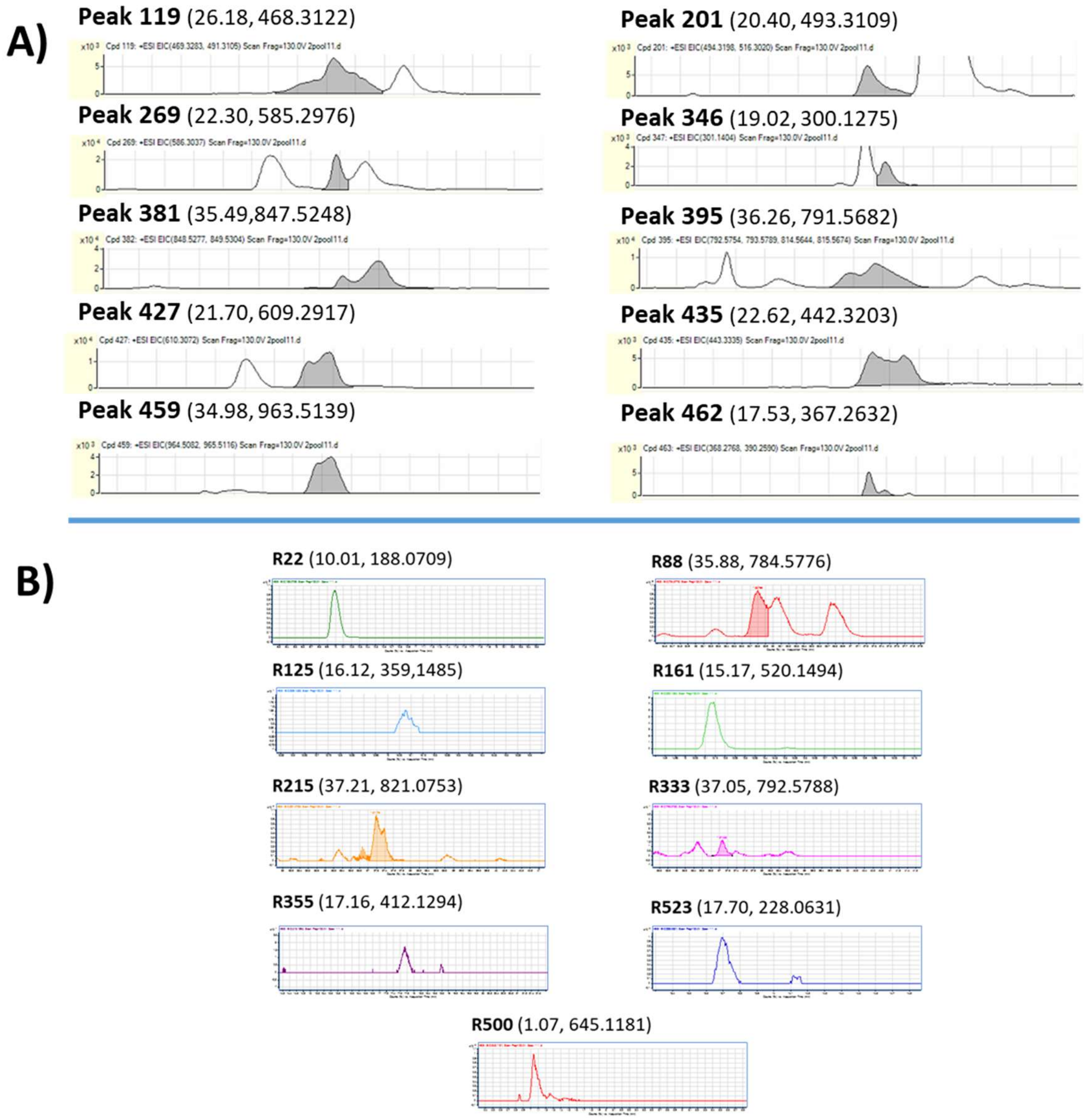

Fig. S2. Chromatograms before and after the alignment step performed by XCMS using the parameters optimized by IPO. In each figure, there are 21 superposed chromatograms from QC samples analysed throughout the three batches.

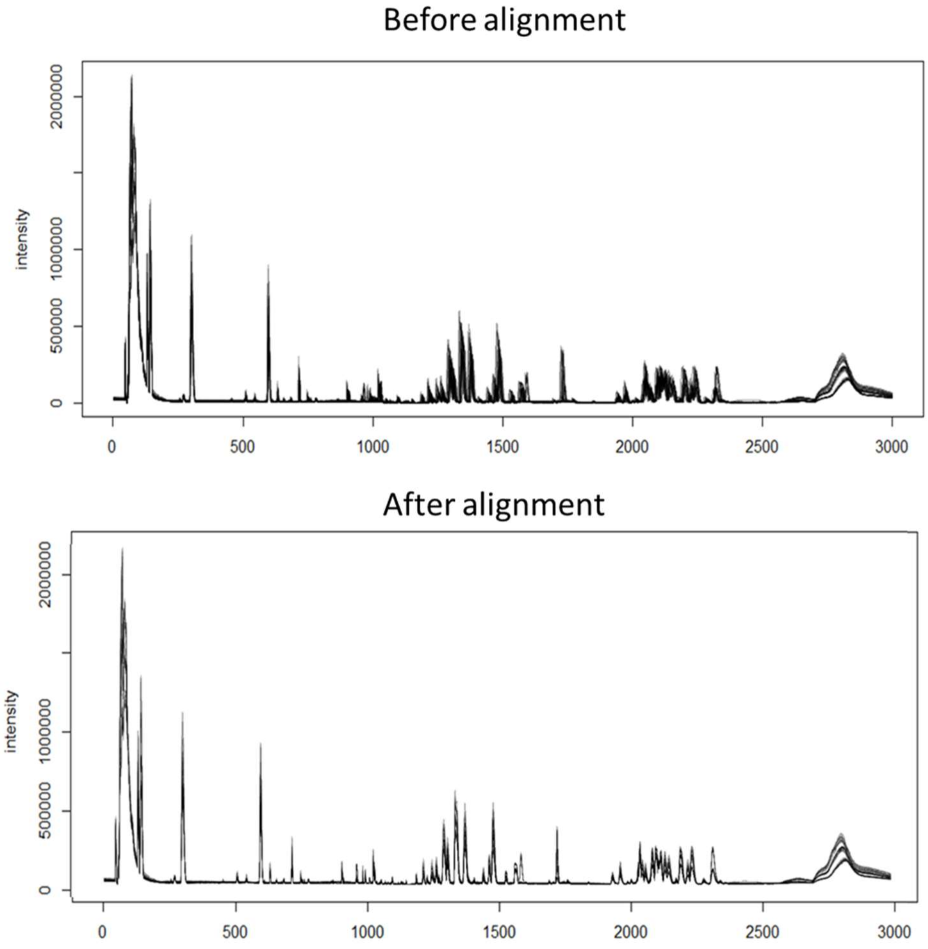

Fig. S3. a) An example of the failure in the automatic area integration step produced using the methodology based on vendor software. It shows how retention times do not change after molecular feature extraction and consequently some peaks are not well integrated. b) The same ion extracted by XCMS c) The result of the example after the alignment of the retention times by XCMS (It shows how retention times change after this step.) c) The result of the example highlighting the section integrated by XCMS. The integration error observed in figure 2a is not observed in this case.

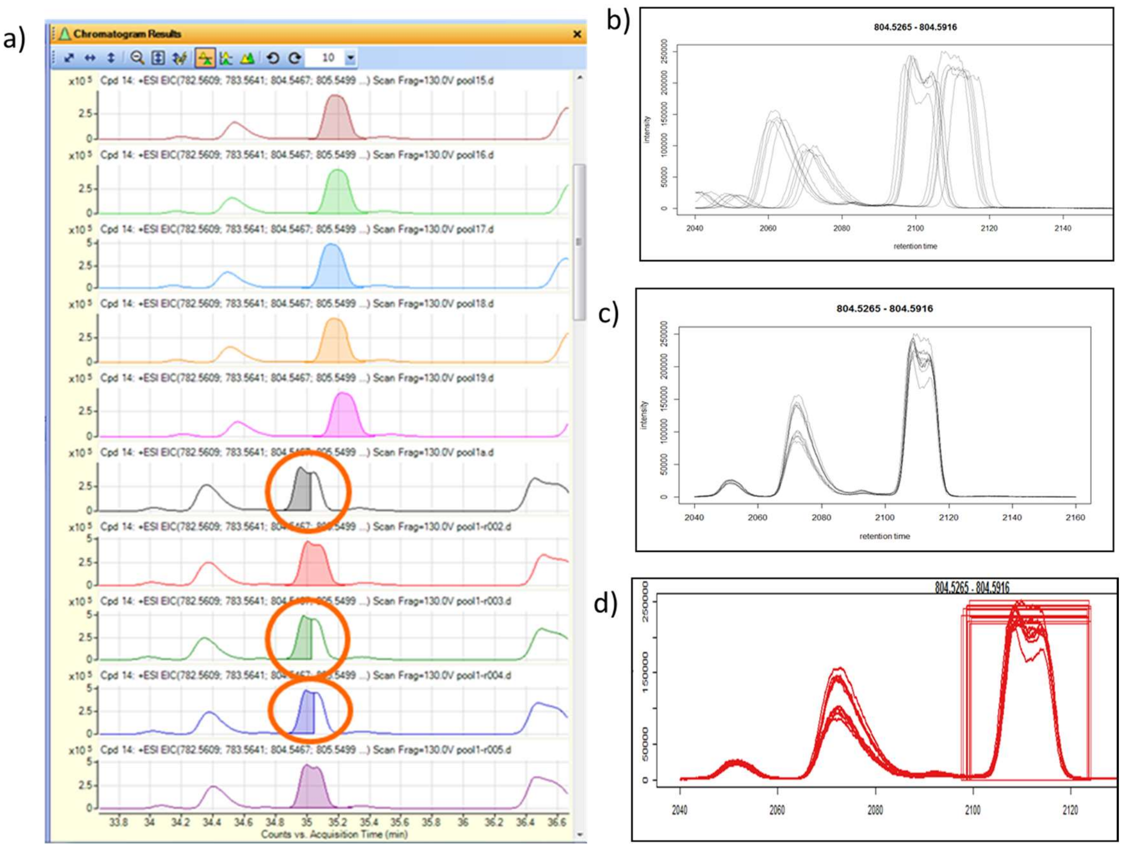

Fig. S4. a) Histogram of the results of the correlations between the peaks areas which were found by both methodologies (*Peak Areas obtained by R versus Peak Areas obtained by Agilent Mass Profinder after manually correction*). **b)** Scatter plots that compares the integrated areas obtained by R and Agilent (after manually correction) of 12 random peaks.

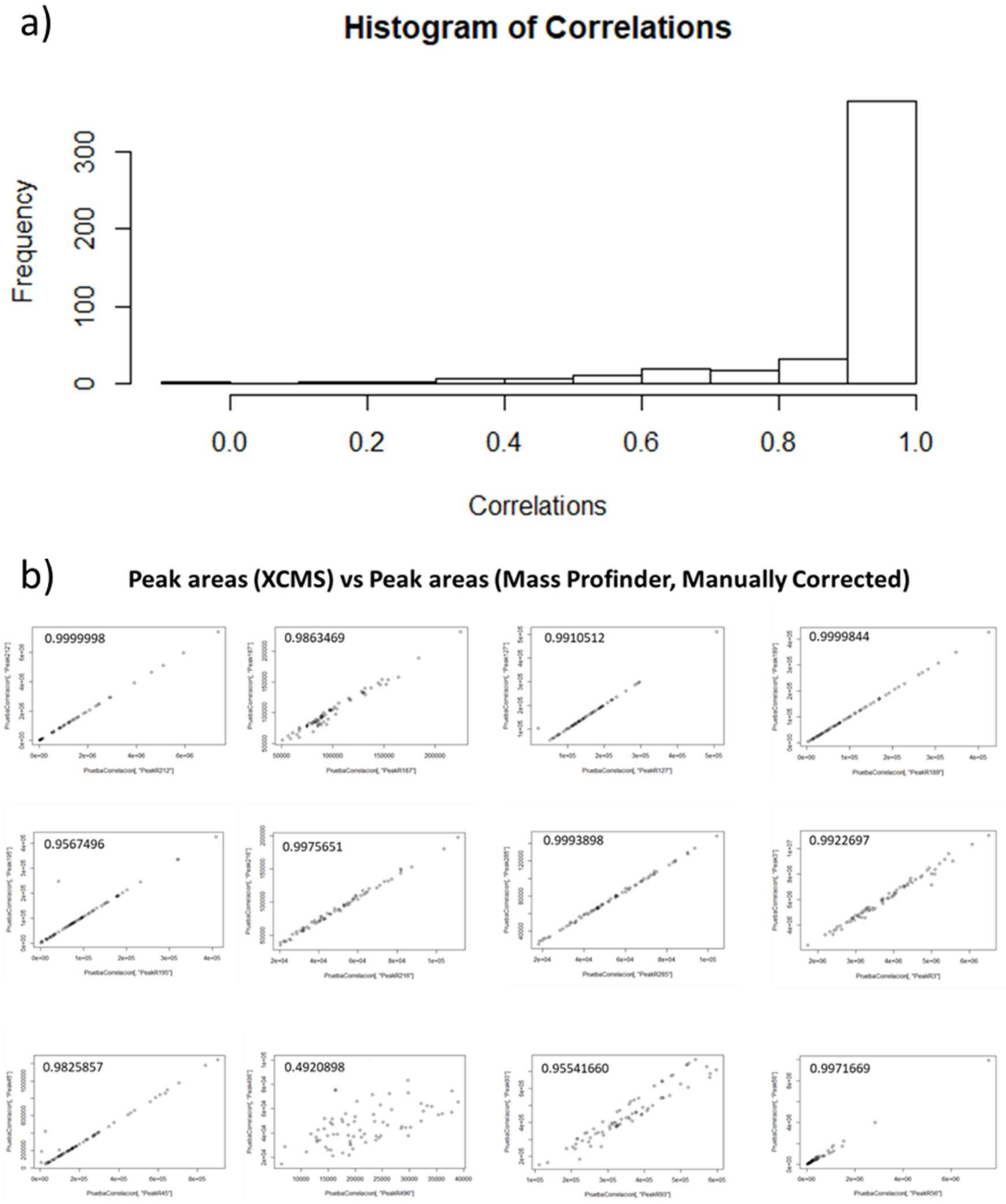

Fig. S5. Histograms of the results of the correlations between the peaks areas which were found by both methodologies (*Peak Areas obtained by R versus Peak Areas obtained by Agilent Mass Profinder before (a) or after (b) manually correction*).

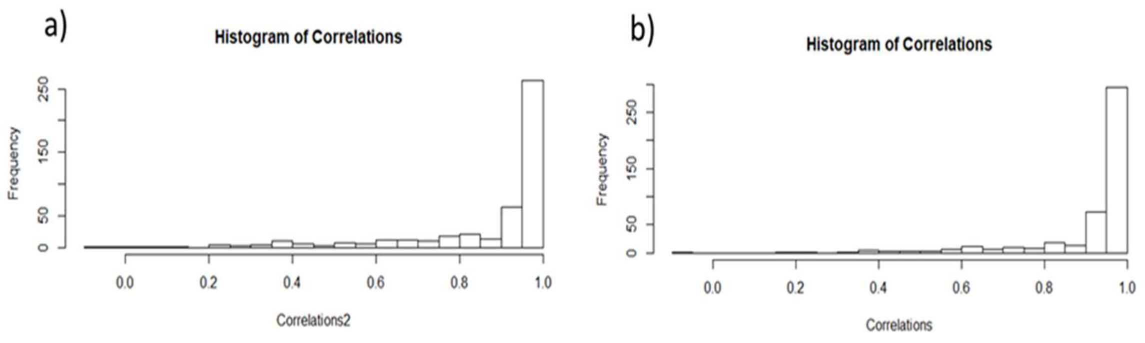

Fig. S6. PCA scores plot from data obtained by methodology based on vendor software. 1a) Raw Data; 1b) data obtained after Bayes method (Metaboanalyst 3.0); 1c) data obtained after bayes and MSTUS methods (Metaboanalyst 3.0) (batch 1, red plots; batch 2, green plots; batch3, blue plots; QCs, inside the circle).

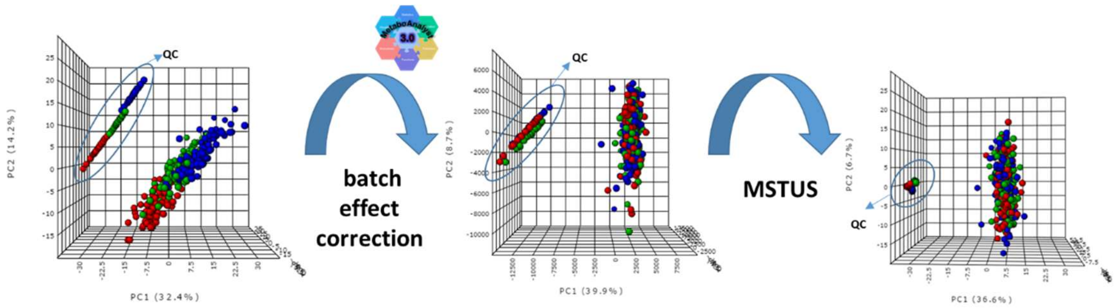

Fig. S7. Examples of different drift trends observed throughout the analytical sequence. These different trends are taken into account in batchCorr normalization.

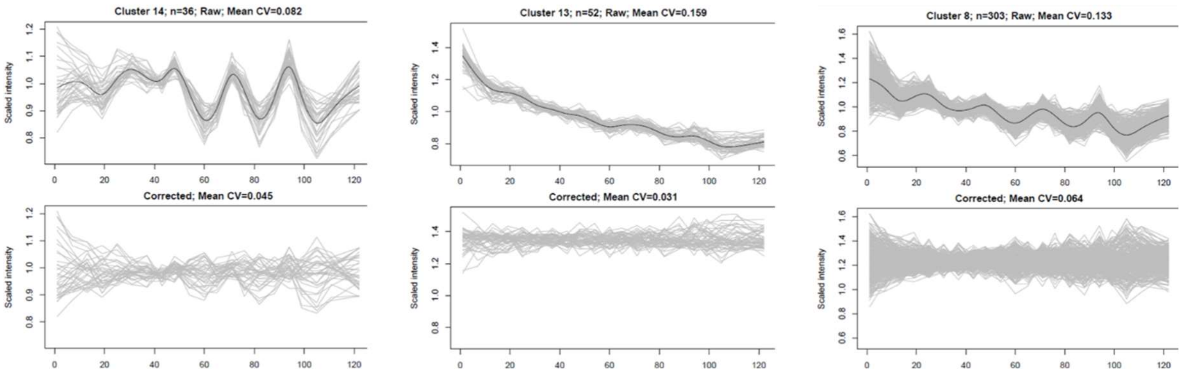

Fig. S8. PCA score plots obtained with data obtained by R methodology. a) Raw data, b) after within batch normalization (batchCorr) c) after between-batch normalization (batchCorr)

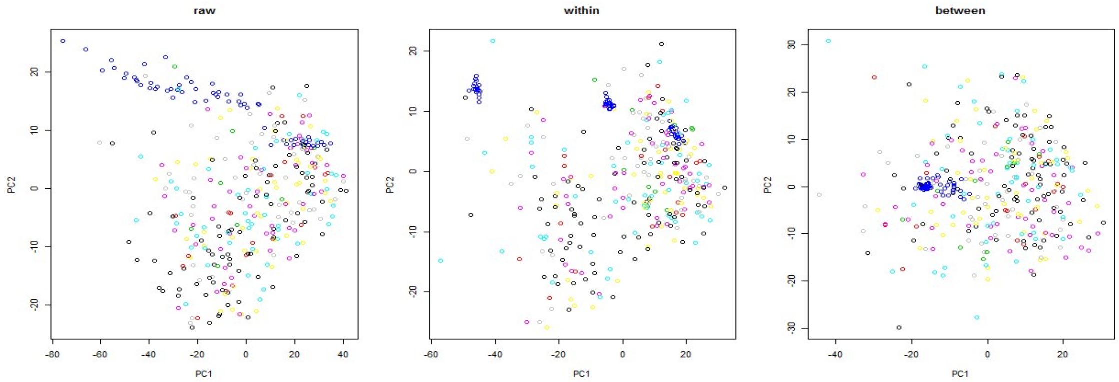

Fig S9. PLS-DA models obtained with the common molecular features extracted by both methodologies. The common significant features in both modes are in bold.

Agilent Profinder Sofware

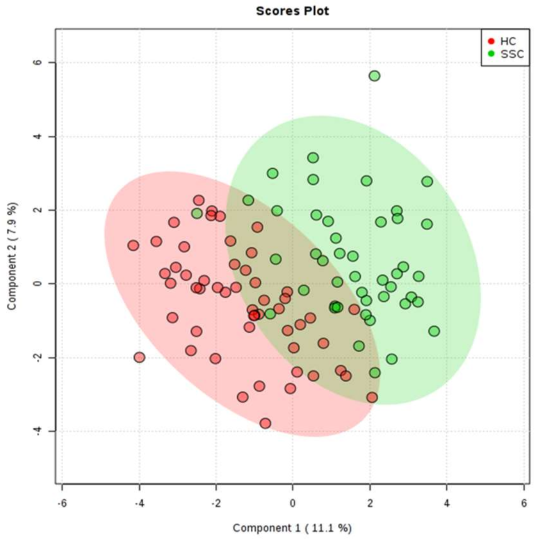

Accuracy: 0.82014, R2 0.60077, Q2:0.36012

15-top compounds: **35**, **61**, **209**, **29**, 401, 30 **52**, **44**, **190**, 198, **53**, **365**, **288**, 422, **91**

R-based methodology

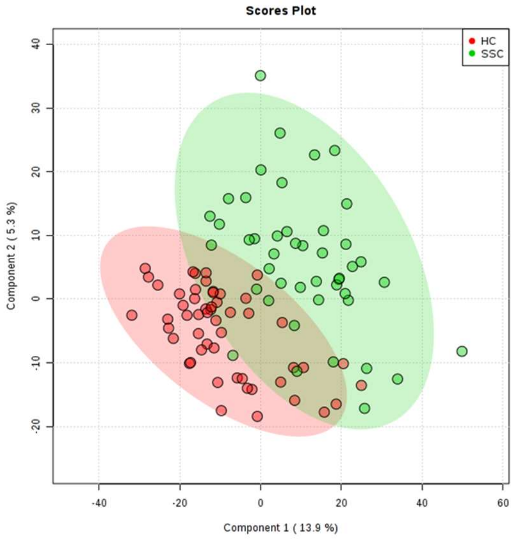

Accuracy: 0.84867, R2 0.64487, Q2:0.47034

15-top compounds: **29**, **52**, **53**, **209**, **35**, **190**, **288**, **91**, **44**, 168, **365**, 48, **61**, 111, 19

Table S1. Detailed analytical information (name, mass, retention time (RT), score, molecular formula and fragments) of the 15 most important peaks in the PLS-DA (Metaboanalyst 3.0) model developed with the data obtained from commercial software.

Peak Nº (Agilent)	Overall Score (%)	Mass	RT (min)	Molecular Formula	Score (%)	Compound name	MS/MS Fragments	Identification Database	Ranking R-model
507	100.0	511.3117	18.20	$C_{24}H_{50}NO_8P$	86.32	PS(O-18:0/0:0)	351.2465/475.2374/534.3116	LMGP03060002	Not extracted
401	85.77	346.0787	13.83	$C_{10}H_{15}N_6O_6P$	77.49	3'-Amino-3'-deoxy-AMP	120.0773/194.0561/222.0507/347.0816	C07026	Not extracted
122	84.10	511.3218	18.40	$C_{24}H_{50}NO_8P$	89.31	PS(O-18:0/0:0)	351.2465/475.2374/534.3116	LMGP03060002	4
127	81.07	208.0857	4.34	$C_{10}H_{12}N_2O_3$	85.66	L-kynurenine	74.0216/94.0627/120.0413/146.0561	HMDB00684	2
61	76.26	180.0650	10.58	$C_7H_8N_4O_2$	97.25	Theophylline/Paraxanthine	55.0283/96.0454/124.0490/181.0805	HMDB01889/ HMDB01860	12
496	65.80	919.5195	34.18	--	--	Unknown	--	--	15
151	63.67	129.0729	1.32	$C_6H_{11}NO_2$	85.83	Pipecolic acid	56.0479/69.0569/84.0804	HMDB01860	5
305	62.39	899.5472	36.26	$C_{49}H_{84}NO_{10}P$	87.42	PS(43:6)	146.9787/649.507/731.4886/773.4973/832.57007	LMGP03010004	28
511	60.98	259.1783	12.78	$C_{13}H_{25}NO_4$	86.61	Hexanoylcarnitine	60.0804/71.0856/85.0287	HMDB00705	30
494	60.69	341.2469	17.14	$C_{19}H_{35}NO_4$	96.48	trans-2-dodecenoylcarnitine	85.0263/163.0386	HMDB13326	17
251	60.33	616.4635	28.76	--	--	Unknown	--	--	10
373	57.60	158.0837	10.01	$C_{10}H_{10}N_2$	86.30	1-Benzylimidazole	159.0837	MetlinID63058	Not extracted
168	56.70	287.2069	15.28	$C_{15}H_{29}NO_4$	96.21	Octanoylcarnitine	60.0785/85.0262/288.2109	HMDB00834	29
91	56.49	315.2366	16.64	$C_{17}H_{33}NO_4$	94.56	Decanoylcarnitine	60.0788/85.0262/316.2425	HMDB62631	22
485	55.31	430.2593	19.05	--	--	Unknown	--	--	Not extracted

Table S2. Detailed analytical information (name, mass, retention time (RT), score, molecular formula and fragments) of the 15 most important peaks in the PLS-DA (Metaboanalyst 3.0) model developed with the data obtained from open source.

Peak Nº (R)	Overall Score (%)	Mass	RT (min)	Molecular Formula	Score (%)	Compound name	MS/MS Fragments	Identification Database	Ranking Vendor-methodology
R_355	100	412.1294	17.75	--	--	Unknown	--	--	Not extracted
R_18	98.58	208.0857	4.33	$C_{10}H_{12}N_2O_3$	85.66	L-kynurenine	74.0216/94.0627/120.0413/146.0561	HMDB00684	4
R_248	95.04	174.0342	10.64	--	--	Unknown	--	--	19
R_75	89.44	511.3259	18.39	$C_{24}H_{50}NO_8P$	89.31	PS(O-18:0/0:0)	351.2465/475.2374/534.3116	LMGP03060002	3
R_52	87.61	378.5454	24.64	$C_{23}H_{38}O_4$	85.09	MG(20:4)	67.0533/81.0685/95.0839	HMDB11578	21
R_223	84.36	129.0792	1.21	$C_6H_{11}NO_2$	85.83	Pipecolic acid	56.0479/69.0569/84.0804/130.0865	HMDB01860	7
R_266	82.29	216.1011	7.92	--	--	Unknown	--	--	Not extracted
R-17	80.40	184.1211	9.01	$C_9H_{16}N_2O_2$	86.53	N-(3-acetamidopropyl)pyrrolidin-2-one	56.9408/86.9471/98.0591/126.0905/185.1262	HMDB61384	18
R_361	80.05	425.3468	19.30	$C_{25}H_{47}NO_4$	72.95	Octadecenoylcarnitine	85.0274/426.3541	HMDB94687	22
R_417	77.91	616.4801	28.40	--	--	Unknown	--		11
R_13	71.55	194.0808	11.98	$C_8H_{10}N_4O_2$	94.86	Caffeine	56.0483/69.0433/83.0593/110.0687/138.064	HMDB01847	32
R_11	71.34	180.0650	10.61	$C_7H_8N_4O_2$	97.25	Theophylline/Paraxanthine	55.0283/96.0454/124.0490/181.0805	HMDB01889/ HMDB01860	5
R_40	70.51	304.2390	26.30	$C_{20}H_{32}O_2$	87.11	Arachiconic acid	55.0530/57.0685/67.0526/71.0842/ 107.0811/121.0995/123.0117/161.112/177.3601	HMDB01043	25
R_414	70.39	596.4926	28.60	$C_{36}H_{68}O_6$	86.75	Glycerol triundecanoate	337.2304/339.2447/361.2273/	HMDB31089	Not extracted
R_486	66.54	919.5195	34.18	--	--	Unknown	--	--	6

Table S3. Detailed analytical information (name, mass, retention time (RT), score, molecular formula and fragments) of the important peaks obtained in the MUVR (PLS) model with the data from R.

Peak № (Agilent)	Rank	Mass	RT (min)	Molecular Formula	Score (%)	Compound Name	MS/MS fragments	Identification Database
PEAK507	3.17	511.3117	18.20	$C_{24}H_{50}NO_8P$	86.32	PS(O-18:0/0:0)	351.2465/475.2374/534.3116	LMGP03060002
PEAK520	5.52	397.3082	18.53	$C_{23}H_{43}NO_4$	77.79	Hexadecanoyl carnitine	85.0286/398.3256	HMDB13207
PEAK367	6.08	934.6784	26.2	$C_{51}H_{99}O_{12}P$	80.66	PI(P-42:0)	Level 3	LMGP06020067
PEAK288	9.03	676.4521	26.4	$C_{36}H_{69}O_9P$	88.88	PG(P-30:1)	Level 3	LMGP04030004
PEAK305	9.35	877.5695	36.26	$C_{49}H_{84}NO_{10}P$	87.42	PS(43:6)	146.9787/649.507/731.4886/773.4973/832.57007	LMGP03010004
PEAK253	9.75	282.2481	26.36	$C_{18}H_{34}O_2$	81.47	Oleic acid	57.0684/69.0686/83.0832/ 97.0984/135.1141/149.1283	HMDB00207
PEAK79	12.72	630.4439	26.36	$C_{35}H_{67}O_7P$	84.47	PA(P-32:1)	57.0685/283.2572	LMGP10030031
PEAK127	13.21	208.0857	4.34	$C_{10}H_{12}N_2O_3$	85.66	L-kynurenine	74.0216/94.0627/120.0413/146.0561	HMDB00684
PEAK227	16.07	361.2158	26.33	$C_{17}H_{34}N_2O_4P$	84.31	Unknown	Level 4	--
PEAK122	17.78	511.3218	18.40	$C_{24}H_{50}NO_8P$	89.31	PS(O-18:0/0:0)	351.2465/475.2374/534.3116	LMGP03060002
PEAK29	18.16	326.2206	26.18	$C_{20}H_{32}O_2$	87.11	Arachiconic acid	55.0530/57.0685/67.0526/71.0842/ 107.0811/121.0995/123.0117/161.112/177.3601	HMDB01043
PEAK414	28.86	436.1598	25.72	$C_{16}H_{28}N_4O_8S$	64.49	Thr Trp Met	Level 3	Metlin16635
PEAK131	29.41	507.3473	24.45	$C_{26}H_{54}NO_6P$	94.21	PC(P-18:0/0:0)	88.1102/508.3473	HMDB13122
PEAK165	32.15	394.1974	26.2	$C_{16}H_{26}N_8O_4$	97.84	Unknown	Level 4	--
PEAK226	34.93	335.2003	25.73	$C_{20}H_{32}O_2P$	84.08	Unknown	Level 4	--
PEAK160	37.45	368.1765	25.73	$C_{14}H_{24}N_8O_4$	97.26	Glycylhystidilargenine	295.1890/369.1924	Metlin21026
PEAK52	39.30	278.2184	25.59	$C_{18}H_{30}O_2$	87.00	Linolenic Acid	69.0660/95.0833/123.1134/279.2177	HMDB01388
PEAK200	48.99	672.5185	30.84	$C_{37}H_{73}N_2O_6P$	71.00	SM(d18:2/14:0)	86.0948/146.9801/512.4402	LMSP03010034
PEAK209	49.16	926.6471	16.05	$C_{45}H_{76}N_{21}O$	96.24	Unknown	Level 4	--
PEAK525	58.03	598.4061	24.66	$C_{26}H_{58}N_6O_9$	97.85	Unknown	Level 4	--
PEAK190	59.13	328.2419	24.37	$C_{22}H_{32}O_2$	83.41	DHA	107.0814/145.0975/173.1304/161.1285	HMDB02183
PEAK312	61.25	733.5548	36.29	$C_{40}H_{80}NO_8P$	81.94	PC(32:0)	Level 3	HMDB07871
PEAK186	64.95	781.5627	35.22	$C_{44}H_{80}NO_8P$	96.43	PC(36:4)	86.0956/146.9812/184.0724/621.4832	LMGP01010927
PEAK30	68.77	302.2129	24.52	$C_{20}H_{30}O_2$	80.91	Eicosapentaenoic acid	81.0686/91.0535/119.0165/303.2146/325.2115	HMDB01999
PEAK264	74.40	549.3545	25.44	$C_{28}H_{56}NO_7P$	94.91	LysoPC(20:1)	86.0939/104.1048/146.9795/367.3151/	HMDB11148
PEAK75	76.36	164.0457	2.39	$C_9H_8O_3$	95.15	m-Coumaric acid	91.0523/119.0471/136.0733/147.0409	HMDB01713

PEAK494	76.53	341.2469	17.14	$C_{19}H_{35}NO_4$	96.48	Trans-2-dodecenoylcarnitine	85.0263/163.0386	HMDB13326
PEAK276	78.42	1537.5468	35.47	--	--	Unknown	--	--
PEAK405	78.55	694.3850	24.65	$C_{26}H_{50}N_{18}O_5$	98.98	Unknown	--	--
PEAK386	80.64	396.2115	28.36	$C_{16}H_{28}N_8O_4$	99.50	Unknown	--	--
PEAK384	82.07	525.2812	21.69	$C_{27}H_{44}NO_7P$	81.45	LysoPE(22:6)	Level 3	HMDB11526
PEAK389	82.42	326.2135	24.35	--	--	Unknown	--	--
PEAK444	83.89	359.2042	24.52	--	--	Unknown	--	--
PEAK347	85.93	1653.0686	35.24	--	--	Unknown	--	--
PEAK401	85.95	346.0787	13.58	$C_{10}H_{15}N_6O_6P$	77.49	3'-Amino-3'-deoxy-AMP	120.0773/194.0561/222.0507/347.0816	C07026
PEAK470	95.41	928.623	24.52	$C_{51}H_{93}O_{12}P$	75.32	PI(O-42:4)	Level 3	LMGP06020069
PEAK224	103.32	369.2780	17.92	$C_{21}H_{39}NO_4$	94.08	cis-5-Tetradecenoylcarnitine	85.0270/370.2902	HMDB02014
PEAK410	103.56	350.2162	24.37	$C_{19}H_{30}N_2O_4$	80.55	Perindopril lactam	351.2251	MetlinID1799
PEAK61	106.74	180.065	10.58	$C_7H_8N_4O_2$	97.25	*Theophylline/Paraxanthine*	55.0283/96.0454/124.0490/181.0805	HMDB01889/ HMDB01860
PEAK511	111.06	259.1783	12.78	$C_{13}H_{25}NO_4$	86.61	Hexanoylcarnitine	60.0804/71.0856/85.0287	HMDB00705
PEAK103	132.69	153.0789	2.39	C_8H_9NO	97.59	Dopamine	65.0370/91.0523/119.0471/136.0731	MetlinID64
PEAK4	136.48	519.3335	21.63	$C_{26}H_{50}NO_7P$	87.88	LysoPC(18:2)	104.1062/184.072	HMDB10386
PEAK166	137.88	515.3059	20.99	$C_{26}H_{46}NO_7P$	81.33	LysoPC(18:3)	104.1060/146.9804/184.0720	HMDB10389
PEAK175	140.23	541.3168	21.26	$C_{28}H_{48}NO_7P$	79.65	LysoPC(20:5)	86.0956/146.9805/184.0718	
PEAK267	142.65	103.0446	1.47	C_4H_9NS	98.39	(±)-2-Methylthiazolidine	56.0481/104.0530	HMDB31682
PEAK144	145.04	517.3666	21.87	$C_{26}H_{48}NO_7P$	60.99	LysoPC(18:3)	96.0954/104.1058/184.0715	HMDB10387
PEAK141	149.88	184.1221	9.05	$C_9H_{16}N2O_2$	86.53	*N*-(*3*-aminopropyl)*pyrrolidin-2-one*	56.9408/86.9471/98.0591/126.0905/185.1262	HMDB61384
PEAK47	161.45	767.5627	36.48	$C_{44}H_{82}NO_7P$	95.32	PC(36:3)	86.0942/146.9794/184.0702	HMDB11246
PEAK373	162.25	158.0837	10.00	$C_{10}H_{10}N_2$	86.30	1-Benzylimidazole	159.0837	MetlinID63058
PEAK379	165.79	775.5092	33.59	$C_{44}H_{74}NO_8P$	77.61	PC(36:7)	86.095/593.4508/717.4421	HMDB08723
PEAK251	166.08	616.4648	28.60	--	--	Unknown	--	--
PEAK298	166.44	468.3006	26.80	$C_{30}H_{44}O_4$	77.96	Unknown	--	--
PEAK169	178.04	700.5492	32.85	$C_{39}H_{77}N_2O_6P$	74.75	SM(d18:1/16:1)	86.0950/184.0715	LMSP03010041

PEAK177	178.38	795.5868	38.85	$C_{46}H_{86}NO_7P$	96.07	PC(37:4)	86.0971/146.9827	HMDB11252
PEAK151	179.63	129.0729	1.32	$C_6H_{11}NO_2$	85.83	Pipecolic acid	56.0479/69.0569/84.0804/130.0865	HMDB01860
PEAK336	180.93	780.5849	38.70	$C_{45}H_{85}N_2O_6P$	97.37	C22:3 Sphingomyelin	Level 3	HMDB13468
PEAK349	183.71	743.5593	36.67	$C_{42}H_{82}NO_7P$	67.14	PC(34:1)	Level 3	HMDB11240
PEAK358	184.03	1273.2775	17.56	--	--	Unknown	--	--
PEAK91	185.86	315.2366	16.64	$C_{17}H_{33}NO_4$	94.56	Decanoylcarnitine	60.0788/85.0262/316.2425	HMDB62631
PEAK168	189.69	287.2069	15.28	$C_{15}H_{29}NO_4$	96.21	Octanoylcarnitine	60.0785/85.0262/288.2109	HMDB00834
PEAK23	191.26	117.0749	1.27	$C_5H_{11}NO_2$	97.78	L-Valine	55.0528/72.0792/117.0749	
PEAK331	192.73	1585.5867	35.25	--	--	Unknown	--	--
PEAK332	194.29	1128.6258	21.61	--	--	Unknown	--	--
PEAK496	194.69	919.5195	34.18	--	--	Unknown	--	--
PEAK528	194.76	253.1263	15.07	$C_{13}H_{19}NO_4$	62.1	3-Indolecarboxylic acid	254.1301	MetlinID6660
PEAK365	197.61	322.1777	23.16	$C_{12}H_{26}N_4O_6$	83.21	Perindoprilat lactam A	323.1926	MetlinID1800
PEAK25	205.07	519.3335	21.63	$C_{26}H_{50}NO_7P$	87.88	LysoPC(18:2)	104.1062/184.072	HMDB10386

Table S4. Details (name, mass, retention time (RT), score, molecular formula and fragments) of the important peaks obtained in the MUVR (PLS) model with the data from R.

Peak Nº (R)	Rank	Mass	RT (min)	Molecular Formula	Score (%)	Compound Name	MS/MS Fragments	Identification Database
R_355	1.52	412.1294	17.76	--	--	Unknown	--	--
R_99	4.50	676.4521	26.35	$C_{51}H_{99}O_{12}P$	80.66	PI(P-42:0)	Level 3	LMGP06020067
R_18	4.79	208.0857	4.33	$C_{10}H_{12}N_2O_3$	85.66	L-kynurenine	74.0216/94.0627/120.0413/146.0561	HMDB00684
R_40	6.69	304.2390	26.35	$C_{20}H_{32}O_2$	87.11	Arachiconic acid	55.0530/57.0685/67.0526/71.0842/ 107.0811/121.0995/123.0117/161.112/177.3601	HMDB01043
R_361	8.25	425.3468	19.32	$C_{21}H_{39}NO_4$	72.95	Octadecenoylcarnitine	85.0274/426.3541	HMDB94687
R_248	9.35	174.0342	10.64	--	--	Unknown	--	--
R_75	14.16	511.3259	18.40	$C_{24}H_{50}NO_8P$	89.31	PS(O-18:0/0:0)	351.2465/475.2374/534.3116	LMGP03060002
R_33	14.56	278.2194	25.72	$C_{18}H_{30}O_2$	87.00	Linolenic Acid	69.0660/95.0833/123.1134/279.2177	HMDB01388
R_36	23.21	672.5185	24.65	$C_{37}H_{73}N_2O_6P$	71.00	SM(d18:2/14:0)	86.0948/146.9801/512.4402	LMSP03010034
R_32	50.79	276.2032	23.72	$C_{18}H_{28}O_2$	60.57	Stearidonic acid	55.0521/69.0687/95.0825/161.9743/	HMDB06547
R_48	52.06	328.2368	24.35	$C_{22}H_{32}O_2$	83.41	Docosahexanoic acid	107.0814/145.0975/173.1304/161.1285	HMDB02183
R_39	57.54	302.2195	24.65	$C_{20}H_{30}O_2$	80.91	Eicosapentaenoic acid	81.0686/91.0535/119.0165/303.2146/325.2115	HMDB01999
R_46	94.75	348.2002	24.49	--	--	Unknown	--	--
R_41	103.28	517.3666	21.87	$C_{26}H_{48}NO_7P$	60.99	LysoPC(18:3)	96.0954/104.1058/184.0715	HMDB10387
R_17	103.43	184.1211	9.04	$C_9H_{16}N_2O_2$	86.53	N-(3-acetamidopropyl)pyrrolidin-2-one	56.9408/86.9471/98.0591/126.0905/185.1262	HMDB61384

Table S5. Members from the Precisesads Clinical Consortium:

Researchers and clinicians	Clinical center
Lorenzo Beretta, Barbara Vigone	Referral Center for Systemic Autoimmune Diseases, Fondazione IRCCS Ca' Granda Ospedale Maggiore Policlinico di Milano, Italy.
Jacques-Olivier Pers, Alain Saraux, Valérie Devauchelle-Pensec, Divi Cornec, Sandrine Jousse-Joulin	Centre Hospitalier Universitaire de Brest, Hospital de la Cavale Blanche, Brest, France.
Bernard Lauwerys, Julie Ducreux, Anne-Lise Maudoux	Pôle de pathologies rhumatismales systémiques et inflammatoires, Institut de Recherche Expérimentale et Clinique, Université catholique de Louvain, Brussels, Belgium.
Carlos Vasconcelos, Ana Tavares, Esmeralda Neves, Raquel Faria, Mariana Brandão	Centro Hospitalar do Porto, Portugal.
Ana Campar, António Marinho, Fátima Farinha, Isabel Almeida	Servicio Cantabro de Salud, Hospital Universitario Marqués de Valdecilla, Santander, Spain.
Miguel Angel Gonzalez-Gay Mantecón, Ricardo Blanco Alonso, Alfonso Corrales Martínez	Servicio Cantabro de Salud, Hospital Universitario Marqués de Valdecilla, Santander, Spain.
Ricard Cervera, Ignasi Rodríguez-Pintó, Gerard Espinosa	Hospital Clinic I Provicia, Institut d'Investigacions Biomèdiques August Pi i Sunyer, Barcelona, Spain.
Rik Lories, Ellen De Langhe	Katholieke Universiteit Leuven, Belgium.
Nicolas Hunzelmann, Doreen Belz	Klinikum der Universitaet zu Koeln, Cologne, Germany.
Torsten Witte, Niklas Baerlecken	Medizinische Hochschule Hannover, Germany.
Georg Stummvoll, Michael Zauner, Michaela Lehner	Medical University Vienna, Vienna, Austria.
Eduardo Collantes, Rafaela Ortega-Castro, Mª Angeles Aguirre-Zamorano, Alejandro Escudero-Contreras, Mª Carmen Castro-Villegas	Servicio Andaluz de Salud, Hospital Universitario Reina Sofía Córdoba, Spain.
Norberto Ortego, María Concepción Fernández Roldán	Servicio Andaluz de Salud, Complejo hospitalario Universitario de Granada (Hospital Universitario San Cecilio), Spain.

Enrique Raya, Inmaculada Jiménez Moleón	Servicio Andaluz de Salud, Complejo hospitalario Universitario de Granada (Hospital Virgen de las Nieves), Spain.
Enrique de Ramon, Isabel Díaz Quintero	Servicio Andaluz de Salud, Hospital Regional Universitario de Málaga, Spain
Pier Luigi Meroni, Maria Gerosa, Tommaso Schioppo, Carolina Artusi,	Università degli studi di Milano, Milan, Italy.
Carlo Chizzolini, Aleksandra Zuber, Donatienne Wynar,	Hospitaux Universitaires de Genève, Switzerland.
Laszló Kovács, Attila Balog, Magdolna Deák, Márta Bocskai, Sonja Dulic, Gabriella Kádár	University of Szeged, Szeged, Hungary.
Falk Hiepe, Velia Gerl, Silvia Thiel	Charite, Berlin, Germany.
Manuel Rodriguez Maresca, Antonio López-Berrio, Rocío Aguilar-Quesada, Héctor Navarro-Linares	Andalusian Public Health System Biobank, Granada, Spain
Yiannis Ioannou, Chris Chamberlain, Jacqueline Marovac.	UCB Pharma, Slough, United Kingdom (PRECISESADS Project office)
Marta Alarcón Riquelme, Tania Gomes Anjos.	Department of Medical Genomics, Center for Genomics and Oncological Research (GENYO), Granada, Spain (PRECISESADS Project Office)
